# Multipartite Correlations in Quantum Collision Models

**DOI:** 10.3390/e24040508

**Published:** 2022-04-05

**Authors:** Sergey Filippov

**Affiliations:** Department of Mathematical Methods for Quantum Technologies, Steklov Mathematical Institute of Russian Academy of Sciences, Gubkina St. 8, 119991 Moscow, Russia; sergey.filippov@mi-ras.ru

**Keywords:** collision model, repeated interactions, quantum correlations, matrix product state, matrix product density operator, tensor network, master equation, memory kernel

## Abstract

Quantum collision models have proved to be useful for a clear and concise description of many physical phenomena in the field of open quantum systems: thermalization, decoherence, homogenization, nonequilibrium steady state, entanglement generation, simulation of many-body dynamics, and quantum thermometry. A challenge in the standard collision model, where the system and many ancillas are all initially uncorrelated, is how to describe quantum correlations among ancillas induced by successive system-ancilla interactions. Another challenge is how to deal with initially correlated ancillas. Here we develop a tensor network formalism to address both challenges. We show that the induced correlations in the standard collision model are well captured by a matrix product state (a matrix product density operator) if the colliding particles are in pure (mixed) states. In the case of the initially correlated ancillas, we construct a general tensor diagram for the system dynamics and derive a memory-kernel master equation. Analyzing the perturbation series for the memory kernel, we go beyond the recent results concerning the leading role of two-point correlations and consider multipoint correlations (Waldenfelds cumulants) that become relevant in the higher-order stroboscopic limits. These results open an avenue for the further analysis of memory effects in collisional quantum dynamics.

## 1. Introduction

**Figure 1:**
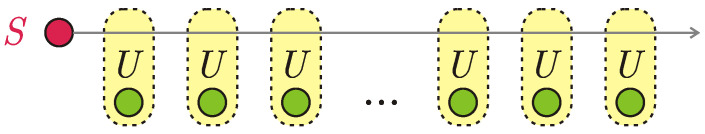
Standard collision model.

The standard collision model, introduced as early as in 1963 in Ref. [1], considers a quantum system that sequentially interacts with identical uncorrelated ancillary particles or oscillator modes. Each elementary system-particle interaction lasts for a finite period of time τ and is described by an elementary unitary evolution operator *U*; see Figure 1.

However simple this model may look like, it (i) naturally describes the system dynamics induced by repeated interactions, e.g., in the micromaser theory [2]; (ii) gives an intuitively clear picture of various phenomena, such as thermalization [3,4], decoherence [4,5,6], homogenization [7,8], nonequilibrium steady state [9,10,11], and entanglement generation [11,12,13]; and (iii) is amenable to analytical treatment, which makes it possible to derive time-continuous master equations in appropriate limits on the system-environment interaction strength and the collision duration [14,15,16] (in the standard collision model, the system dynamics are Markovian and completely positive divisible due to a past-future independence of ancillary particles [17]). Ideas of repeated interactions underlie the discrete-time open quantum walks and their continuous-time limit [18,19,20,21,22,23]. Hence, it is no wonder that quantum collision models are becoming increasingly popular in quantum information, quantum technology, and mathematical physics communities. Mysteriously, the quantum physics community and mathematical physics community do not know much about each other and sometimes conduct rather isolated research on highly interrelated topics. Mathematical physicists usually refer to the standard quantum collision model as the repeated interaction model and treat it as a particular model of non-equilibrium quantum statistical mechanics [24]. In addition to the derivation of the master equation, the interest of mathematical physicists is also focused on the asymptotic state in the limit of large times [25,26] and the study of random repeated interactions [27,28]. On the other hand, quantum physicists find new applications of quantum collision models in simulations of open quantum many-body dynamics [29,30] (including simulations on noisy intermediate-scale quantum processors [31]), relaxation processes caused by the dilute gas environment [32], quantum thermodynamics [33], and quantum thermometry [34,35]. The collisional picture of repeated interactions also takes place in quantum optics and waveguide quantum electrodynamics, where the electromagnetic field is represented in the form of discrete time-bin modes interacting with a quantum emitter [36,37,38,39,40,41,42,43,44,45,46,47,48,49]; however, the time-bin modes constituting the radiation field can be correlated so that the system dynamics becomes non-Markovian and exhibits memory effects in general. Besides the initially correlated state of ancillary particles or modes [46,47,48,49,50,51], memory effects in quantum collision models also appear as a result of two-ancilla collisions in between the system-ancilla collisions, where the latest involved ancilla interacts with the one that would interact with the system during the next collision [52,53,54,55]. An alternative scenario for non-Markovian dynamics (e.g., due to random telegraph noise) assumes that a system is composed of the very open system under study and an auxiliary sybsystem, which alternately interacts with a fresh reservoir ancilla and the system under study [56]. Another approach considers repeated interactions of the system with the particles it has already collided (including many-body collisions) [57,58,59]. Quantum channels with memory can also be viewed in terms of quantum collision models [60,61,62,63,64,65,66,67]. The presented list of possible modifications for quantum collision models is far from being complete; in this regard, we refer the interested reader to the recent review papers [68,69]. Nonetheless, the reader can see a great flexibility of quantum collision models to describe a variety of physical situations in a rather simple way.

One of the current challenges in the standard collision model is related to quantum correlations among ancillas that are induced by successive system-ancilla interactions. These correlations lead to an advantage in the collisional quantum thermometry [34]. However, a direct numerical simulation of the output ancillas’ state is possible for a relatively small number *n* of ancillas because of an exponentially growing dimension, $d^{n}$, for the state of *d*-dimensional ancillas. This includes, for instance, d=2 and n≤12 in Ref. [34]. Another challenge appears if the ancillas are initially correlated. This scenario takes place, e.g., when the second system starts interacting with an array of ancillas that were originally uncorrelated but previously interacted with the first system in the standard collision model [65,66]. Alternatively, the ancillas can represent time-bin correlated modes in the structured electromagnetic radiation [36,37,38,39,40,41,42,43,44,45,46,47,48,49] or particles in a correlated spin chain, e.g., spin-1 particles in the ground state of the Affleck–Kennedy–Lieb–Tesaki (AKLT) antiferromagnetic Hamiltonian [70]. Ref. [71] reports that the correlations can break convergence of the system state to the same state of all locally identical ancillas (such a convergence—known as homogenization—would have taken place under appropriate conditions, were the ancillas uncorrelated). Again, the exponential increase in Hilbert-space dimension limits the numerical study in Ref. [71] to 16 ancillas. Therefore, we face a general problem of how to deal with correlations among ancillas (either induced by the system or initially present).

The first goal of this paper is to represent the system-induced correlations among ancillas (in the standard collision model) by developing the tensor network formalism applied recently in Ref. [72]. The main idea behind the tensor network representation (in the form of the matrix product state [73,74,75,76]) is that many *n*-partite states of *d*-dimensional ancillas require only about $ndr^{2}$ complex parameters to be specified, not $d^{n}$ parameters. As we show in this paper, *r* equals the system dimension in the standard collision model. Our second goal is to develop the ideas of Ref. [72] and derive a more general master equation for the system dynamics in the nonstandard collision model with an initially correlated environment. The point of Ref. [72] is that two-point correlations among ancillas play a leading role in the system dynamics if each elementary unitary evolution operator slightly deviates from the identity operator. However, it may happen that the leading contribution vanishes for a specific interaction, and we demonstrate such an example in this paper. Therefore, one needs to consider higher-order correlations among ancillas and their effect on the system dynamics. We close this gap and provide a recipe for how to derive a master equation valid in the corresponding perturbation order for the elementary unitary evolution operator.

## 2. Tensor Network Notation

Tensor network representation of quantum states is reviewed in a number of papers [73,74,75,76,77,78] and a book [79]. Consider a pure state |ψ〉 of *n* particles, where each particle is associated with a Hilbert space H of a finite dimension *d*. The state is fully defined by $d^{n}$ complex numbers $C_{i_{1}i_{2}\ldots i_{n}}$ in the decomposition

(1) $$|\psi \rangle =\sum_{i_{1},i_{2},\ldots ,i_{n}=1,\ldots ,d}C_{i_{1}i_{2}\ldots i_{n}}|i_{1}\rangle ⊗|i_{2}\rangle ⊗\ldots ⊗|i_{n}\rangle ,$$

where ${\left\{|i_{k}\rangle \right\}}_{i_{k}=1,\ldots ,d}$ is an orthonormal basis in H. A collection of $d^{n}$ complex numbers $\left\{C_{i_{1}i_{2}\ldots i_{n}}\right\}$ can be viewed as a rank-*n* tensor *C* with a picture representation involving a letter “*C*” with *n* legs. To distinguish the ket-vector |ψ〉 from the bra-vector 〈ψ|, we add arrows to the legs; namely, we associate outcoming arrows with ket-vectors and incoming legs with bra-vectors.

**Figure 2:**
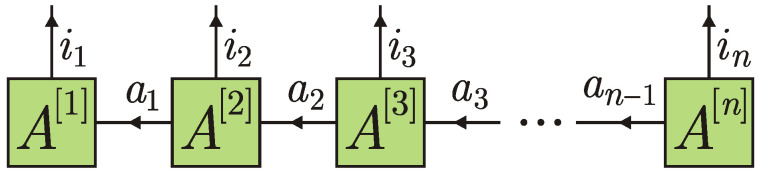
Tensor diagram for a matrix product state.

A tensor diagram concisely depicts a contraction of tensors: the connected lines are summed over. The tensor diagram for an *n*-partite matrix product state (MPS) with open boundary conditions contains *n* tensors $A^{\left[1\right]},\ldots ,A^{\left[n\right]}$ connected in a line; see Figure 2.

$A^{\left[1\right]}$ and $A^{\left[n\right]}$ are rank-2 tensors with elements $A_{a_{1}}^{\left[1\right],i_{1}}$ and $A_{a_{n-1}}^{\left[n\right],i_{n}}$, respectively, whereas for all k=2,…,n−1 the tensor $A^{\left[k\right]}$ has rank 3 and is composed of elements $A_{a_{k-1},a_{k}}^{\left[k\right],i_{k}}$. On the other hand, if the physical index $i_{k}$ is fixed, then $A^{\left[k\right],i_{k}}$ can be viewed as a matrix with elements $A_{a_{k-1},a_{k}}^{\left[k\right],i_{k}}$. Similarly, if $i_{1}$ and $i_{n}$ are fixed, then $A^{\left[1\right],i_{1}}$ and $A^{\left[n\right],i_{n}}$ can be viewed as a row matrix and a column matrix with matrix elements $A_{1,a_{1}}^{\left[1\right],i_{1}}$ and $A_{a_{n-1},1}^{\left[n\right],i_{n}}$, respectively. Arrows in Figure 2 also indicate the order for multiplication of matrices. The contraction yields

(2) $$C_{i_{1}i_{2}\ldots i_{n}}=A^{\left[1\right],i_{1}}A^{\left[2\right],i_{2}}\cdots A^{\left[n\right],i_{n}},$$

which explains the MPS name. A number $\left|\{a_{k}\}\right|$ of the values that the virtual index $a_{k}$ can take is not related to the physical dimension *d* of the particles. We will refer to the maximal number $\max_{k=1,\ldots ,n-1}\left|\left\{a_{k}\right\}\right|$ as the bond dimension. Clearly, the MPS representation for a given state |ψ〉 is not unique in general; however, the less the bond dimension, the easier the calculations and the analysis. In view of this, the minimal bond dimension among all possible MPS representations is called the MPS rank and denoted by *r*. The greater *r*, the more entangled the state |ψ〉 can be with respect to the left-right bipartitions [80].

**Figure 3:**
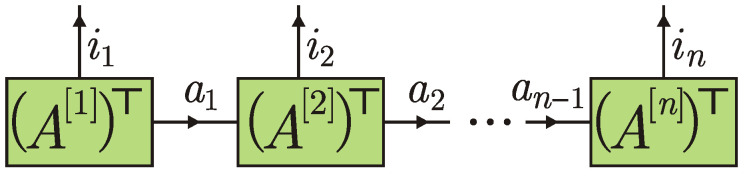
Equivalent diagram to that in Figure 2. ⊤ denotes transposition with respect to virtual indices.

Arrows in tensor diagrams simplify their interpretation. For instance, changing the direction of arrows from left to right in the connecting lines in Figure 2, we get the transposed matrices ${\left(A^{\left[k\right],i_{k}}\right)}^{⊤}$, k=1,…,n. The resulting diagram is depicted in Figure 3.

Nonetheless, if indices $i_{1},\ldots ,i_{n}$ are fixed, then the c-number $C_{i_{1}i_{2}\ldots i_{n}}$ does not change because

(3) $$C_{i_{1}i_{2}\ldots i_{n}}\equiv \underset{1\times 1\;\mathrm{matrix}}{\left(C_{i_{1}i_{2}\ldots i_{n}}\right)}={\left(C_{i_{1}i_{2}\ldots i_{n}}\right)}^{⊤}={\left(A^{\left[n\right],i_{n}}\right)}^{⊤}\cdots {\left(A^{\left[2\right],i_{2}}\right)}^{⊤}{\left(A^{\left[1\right],i_{1}}\right)}^{⊤}.$$

## 3. Matrix Product State Correlations in the Standard Collision Model

**Figure 4:**
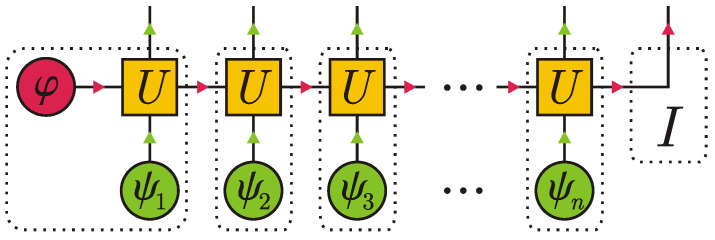
Matrix product state of the system and ancillas induced by collisions in the standard collision model.

We begin with the simplest scenario, in which the system is initially in a pure state $|\varphi \rangle \in H_{S}$, $\dim H_{S}=d_{S}$, and the environment consists of *n* uncorrelated ancillas in a pure state $|\psi_{1}\rangle ⊗|\psi_{2}\rangle ⊗\ldots ⊗|\psi_{n}\rangle \in H^{⊗n}$, dimH=d. Each elementary collision is described by a unitary operator $U:H_{S}⊗H\to H_{S}⊗H$, which is viewed as a 4-rank tensor. After *n* collisions, the system and ancillas get entangled, and their composite state is given by a tensor diagram in Figure 4.

As a result of *n* collisions, we get a correlated state of n+1 particles: *n* ancillas and one system particle. Dotted lines in Figure 4 denote tensors that should be contracted to get the matrix product state structure. The rightmost dotted region depicts an identity operator *I*. Clearly, the bond dimension equals the number of the system degrees of freedom, $d_{S}$. Therefore, we can associate each virtual index $a_{k}$ with a vector $|a_{k}\rangle \in H_{S}$, so that a collection of vectors $\left\{|a_{k}\rangle \right\}$ for a fixed *k* forms an orthonormal basis in $H_{S}$. The very diagram in Figure 4 serves as the proof for the following result.

**Proposition** **1.** *Let the system and n ancillas be initially in the pure states |φ〉, $|\psi_{1}\rangle$, …, $|\psi_{n}\rangle$. Then the output state $|\Psi \rangle \in H^{⊗n}⊗H_{S}$ of the system and ancillas in the standard collision model with the elementary unitary operator U adopts an MPS representation*

(4) $$|\Psi \rangle =\sum_{i_{1},i_{2},\ldots ,i_{n}=1}^{d}\sum_{i_{n+1}=1}^{d_{S}}A^{\left[1\right],i_{1}}A^{\left[2\right],i_{2}}\cdots A^{\left[n\right],i_{n}}A^{\left[n+1\right],i_{n+1}}|i_{1}\rangle ⊗|i_{2}\rangle ⊗\ldots ⊗|i_{n}\rangle ⊗|i_{n+1}\rangle ,$$

*where $A_{1,a_{1}}^{\left[1\right],i_{1}}=\langle a_{1}|⊗\langle i_{1}|U|\varphi \rangle ⊗|\psi_{1}\rangle$, $A_{a_{k-1},a_{k}}^{\left[k\right],i_{k}}=\langle a_{k}|⊗\langle i_{k}|U|a_{k-1}\rangle ⊗|\psi_{k}\rangle$ for all k=2,…,n, and $A_{a_{n},1}^{\left[n+1\right],i_{n+1}}=\delta_{a_{n},i_{n+1}}$.*

The result of Proposition 1 explains the previously known observations of Ref. [8] that the partial swap interactions ($U=\exp[-ig\tau \sum_{i,j}|ij\rangle \langle ji|]$) generate the *W*-type of entanglement, whereas the controlled unitary interactions ($U=\sum_{i}U_{i}⊗|i\rangle \langle i|$) generate entanglement of the Greenberger–Horne–Zeilinger (GHZ) type. In fact, both *W* and GHZ states of many qubits are particular forms of the matrix product states with the bond dimension 2 [73,74,75,76,77,78].

**Example** **1.** *Let the system and ancillas be qubits. The system is initially in the excited state |φ〉=|↑〉. Each ancilla is initially in the ground state, i.e., $|\psi_{k}\rangle =|↓\rangle$ for all k. Consider the energy exchange unitary U=exp[gτ(|↓↑〉〈↑↓|−|↑↓〉〈↓↑|)]. Then, Proposition 1 yields*

$$\begin{array}{ccc} A^{[1],↓}=\left(\begin{array}{cc} 0 & \cos g\tau \end{array}\right),\;A^{[k],↓}=\left(\begin{array}{cc} 1 & 0\\ 0 & \cos g\tau \end{array}\right)\;\mathrm{for}\;k=2,\ldots ,n,\;A^{[n+1],↓}=\left(\begin{array}{c} 1\\ 0 \end{array}\right), & & \\ A^{[1],↑}=\left(\begin{array}{cc} \sin g\tau & 0 \end{array}\right),\;A^{[k],↑}=\left(\begin{array}{cc} 0 & 0\\ \sin g\tau & 0 \end{array}\right)\;\mathrm{>for}\;k=2,\ldots ,n,\;A^{[n+1],↑}=\left(\begin{array}{c} 0\\ 1 \end{array}\right). & \end{array}$$

*Note that the matrix $A^{[k],↑}$ is nilpotent, i.e., the product of the matrix with itself is equal to a null matrix. For this reason, $A^{[k],↑}A^{[k+1],↑}=0$, which means that after interactions, the adjacent ancillas cannot be in the state |↑〉. Similarly, $A^{[k],↑}\left(\prod_{l=1}^{m-1}A^{[k+l],↓}\right)A^{[k+m],↑}=0$, which shows that any two ancillas cannot simultaneously occupy the state |↑〉. The system and ancillas are finally in the W-like state*

$$|\Psi \rangle =\sum_{k=0}^{n-1}\cos^{k}g\tau \sin g\tau |\underset{k}{\underset{︸}{↓\ldots ↓}}↑\underset{n-k}{\underset{︸}{↓\ldots ↓}}\rangle +\cos^{n}g\tau |\underset{n}{\underset{︸}{↓↓\ldots ↓↓}}↑\rangle .$$

The explicit relation between the unitary operator *U* and tensors $A^{\left[k\right]}$, which we establish in Proposition 1, enables one to approach the quantum engineering problem too. Suppose one wants to create an entangled state $|\Psi^{\prime }\rangle$ of *n* particles that adopts a matrix product representation with the bond dimension *r*. Then one needs to take an *r*-dimensional quantum system and let it sequentially interact with the initially uncorrelated particles. Finally, one performs a projective measurement on the system in the basis $\left\{|i_{n+1}\rangle \right\}$ to get rid of its degrees of freedom. The resulting state of *n* particles is $\langle i_{n+1}^{\prime }|\Psi \rangle$, where $i_{n+1}^{\prime }$ is the measurement outcome and |Ψ〉 is given by Equation (4). The unitary operator *U* should be optimized in such a way as to maximize the overlap ${\left|\left(\langle \Psi^{\prime }|⊗\langle i_{n+1}^{\prime }|\right)|\Psi \rangle \right|}^{2}$. Clearly, each collision could be described by its own unitary operator. Then, one should replace $U\to U_{k}$ in the formula for $A^{\left[k\right]}$ in Proposition 1. Numerical tools for optimization over many unitary operators ${\left\{U_{k}\right\}}_{k=1}^{n}$ are presented, e.g., in Refs. [81,82].

**Figure 5:**
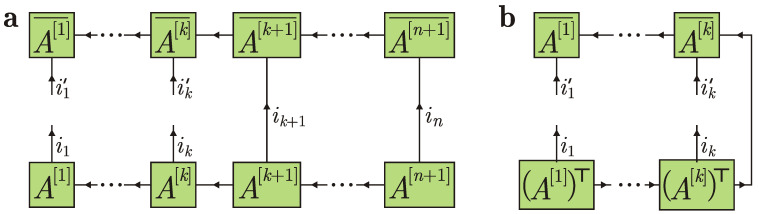
(**a**) Tensor diagram for the reduced density operator $\varrho_{1\ldots k}$. Overline denotes complex conjugation. (**b**) Simplified tensor diagram for $\varrho_{1\ldots k}$ due to the right normalization condition.

Let us consider entanglement of the state |Ψ〉 with respect to a bipartition into ancillas 1,…,k on one side and ancillas k+1,…,n and the system on the other side, i.e., the left-right bipartition with the boundary in between the ancillas *k* and k+1. Entanglement of a pure state with respect to a bipartition is quantified by the entanglement entropy that equals the von Neumann entropy of either reduced density operator, $S\left(\varrho_{1\ldots k}\right)=S\left(\varrho_{k+1\ldots nS}\right)$, where $S\left(\varrho \right)=-\mathrm{tr}\left[\varrho \log_{2}\varrho \right]$. The reduced density operator $\varrho_{1\ldots k}={\mathrm{tr}}_{k+1,\ldots ,n+1}|\Psi \rangle \langle \Psi |$ for *k* ancillas is presented in the form of a tensor diagram in Figure 5a.

The tensor diagram in Figure 5a gets simpler if we take into account the following important property (referred to as the right-normalization condition [75]):

(5) $$\sum_{i_{m},a_{m}}A_{a_{m-1},a_{m}}^{\left[m\right],i_{m}}\bar{A_{a_{m},a_{m-1}^{\prime }}^{\left[m\right],i_{m}}}=\delta_{a_{m-1},a_{m-1}^{\prime }}\Leftrightarrow \sum_{i_{m}}A^{\left[m\right],i_{m}}{\left(A^{\left[m\right],i_{m}}\right)}^{†}=I\;\mathrm{for}\;\mathrm{all}\;m=1,\ldots ,n+1.$$

Here, the overline denotes the complex conjugation and † denotes the Hermitian conjugation. In fact, if m=n+1, then $\sum_{i_{n+1}}A_{a_{n},1}^{\left[n+1\right],i_{n+1}}\bar{A_{1,a_{n}^{\prime }}^{\left[n+1\right],i_{n+1}}}=\delta_{a_{n}a_{n}^{\prime }}$ because $A_{a_{n},1}^{\left[n+1\right],i_{n+1}}=\delta_{a_{n},i_{n+1}}$ by Proposition 1. If m=2,…,n, then Proposition 1 implies

(6) $$\begin{array}{ccc} \sum_{i_{m},a_{m}}A_{a_{m-1},a_{m}}^{\left[m\right],i_{m}}\bar{A_{a_{m},a_{m-1}^{\prime }}^{\left[m\right],i_{m}}} & = & \sum_{i_{m},a_{m}}\bar{A_{a_{m},a_{m-1}^{\prime }}^{\left[m\right],i_{m}}}A_{a_{m-1},a_{m}}^{\left[m\right],i_{m}}\\ & = & \sum_{i_{m},a_{m}}\langle a_{m-1}^{\prime }|⊗\langle \psi_{m}|U^{†}\left(|a_{m}\rangle ⊗|i_{m}\rangle \right)\left(\langle a_{m}|⊗\langle i_{m}|\right)U|a_{m-1}\rangle ⊗|\psi_{m}\rangle \\ & = & \langle a_{m-1}^{\prime }|⊗\langle \psi_{m}|\underset{I}{\underset{︸}{U^{†}U}}|a_{m-1}\rangle ⊗|\psi_{m}\rangle \\ & = & \langle a_{m-1}^{\prime }|a_{m-1}\rangle \langle \psi_{m}|\psi_{m}\rangle \\ & = & \delta_{a_{m-1},a_{m-1}^{\prime }}. \end{array}$$

If m=1, then we deal with dummy indices $a_{0}=a_{0}^{\prime }=1$ and $\sum_{i_{1},a_{1}}A_{1,a_{1}}^{\left[1\right],i_{1}}\bar{A_{a_{1},1}^{\left[1\right],i_{1}}}=\langle \varphi |\varphi \rangle \langle \psi_{1}|\psi_{1}\rangle =1$. Hence, we have proved the following result.

**Proposition** **2.** *MPS |Ψ〉 in Equation (4) satisfies the right-normalization condition (5).*

An MPS satisfying the right normalization condition is also called right-canonical [75]. The advantage of the right-canonical form is that the partial trace over the rightmost particles corresponds to a single connecting line in the tensor diagram; see Figure 5b. Indeed, Equation (5) is equivalent to $\sum_{i_{m}}{\left(A^{\left[m\right],i_{m}}\right)}^{⊤}\bar{A^{\left[m\right],i_{m}}}=I$, which is exactly the vertical connecting line in Figure 5b. Physically, the reduced density operator for *k* ancillas does not depend on future system collisions with other ancillas that happen after time kτ.

Entanglement entropy E(Ψ) of the state |Ψ〉 with respect to the cut in between the ancillas *k* and k+1 reads

(7) $$\begin{array}{ccc} E(\Psi ) & = & S(\varrho_{1\ldots k})\\ & = & S\left(\sum_{i_{1},\ldots ,i_{k},i_{1}^{\prime },\ldots ,i_{k}^{\prime }}\bar{A^{\left[1\right],i_{1}}}\cdots \bar{A^{\left[k\right],i_{k}}}{\left(A^{\left[k\right],i_{k}}\right)}^{⊤}\cdots {\left(A^{\left[1\right],i_{1}}\right)}^{⊤}|i_{1}\rangle \langle i_{1}^{\prime }|⊗\cdots |i_{k}\rangle \langle i_{k}^{\prime }|\right)\\ & = & S\left(\sum_{i_{1},\ldots ,i_{k},i_{1}^{\prime },\ldots ,i_{k}^{\prime }}A^{\left[1\right],i_{1}}\cdots A^{\left[k\right],i_{k}}{\left(A^{\left[k\right],i_{k}}\right)}^{†}\cdots {\left(A^{\left[1\right],i_{1}}\right)}^{†}|i_{1}\rangle \langle i_{1}^{\prime }|⊗\cdots |i_{k}\rangle \langle i_{k}^{\prime }|\right). \end{array}$$

Note that $E\left(\Psi \right)\leq \log d_{S}$ because $d_{S}$ is an upper bound for the Schmidt rank of |Ψ〉.

## 4. Generalization to Mixed States of the System and Ancillas

**Figure 6:**
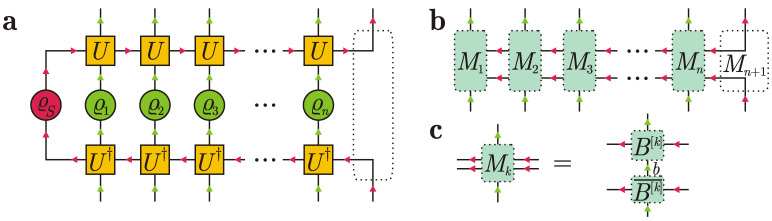
(**a**) Tensor diagram for Equation (8). (**b**) Matrix product density operator. (**c**) Tensor decomposition guaranteeing positive semidefiniteness of the matrix product density operator.

Let us consider the standard collision model, where the system and ancillas are generally mixed. This scenario is especially relevant to the task of quantum thermometry [34,35]. The initial state of the system is given by the density operator $\varrho_{S}$. The initial state of *n* ancillas is given by a factorized density operator ${⨂}_{k=1}^{n}\varrho_{k}$. Collisional dynamics with the elementary unitary operator *U* drives the system and ancillas to the state

(8) $$U_{Sn}\cdots U_{S1}\left(\varrho_{S}⊗\varrho_{1}⊗\ldots ⊗\varrho_{n}\right)U_{S1}^{†}\cdots U_{Sn}^{†},$$

where the subscript Sk in the notation $U_{Sk}$ means that *U* nontrivially acts on the system and the *k*-th ancilla. A tensor diagram for Equation (8) is presented in Figure 6a.

Dotted regions in Figure 6a show tensor contractions or tensor combinations that effectively lead to the equivalent tensor diagram depicted in Figure 6b. Note, however, that the arrows in the upper horizontal line in Figure 6a,b are different. The operator in Figure 6b reads

(9) $$\varrho_{1\ldots nS}\left(n\tau \right)=\sum_{i_{1},\ldots ,i_{n},i_{n+1},i_{1}^{\prime },\ldots ,i_{n}^{\prime },i_{n+1}^{\prime }}M_{1}^{i_{1}i_{1}^{\prime }}\cdots M_{n}^{i_{n}i_{n}^{\prime }}M_{n+1}^{i_{n+1}i_{n+1}^{\prime }}|i_{1}\ldots i_{n}i_{n+1}\rangle \langle i_{1}^{\prime }\ldots i_{n}^{\prime }i_{n+1}^{\prime }|.$$

Here, $M_{1}$ and $M_{n+1}$ are rank-4 tensors, whereas $M_{k}$ is a rank-6 tensor for all k=2, …, *n*. If indices $i_{1}$ and $i_{1}^{\prime }$ are fixed, then we treat $M_{1}^{i_{1}i_{1}^{\prime }}$ as a row matrix with elements ${\left(M_{1}^{i_{1}i_{1}^{\prime }}\right)}_{11,a_{1}a_{1}^{\prime }}$. Similarly, if indices $i_{n+1}$ and $i_{n+1}^{\prime }$ are fixed, then we treat $M_{n+1}^{i_{n+1}i_{n+1}^{\prime }}$ as a column matrix with elements ${\left(M_{n+1}^{i_{n+1}i_{n+1}^{\prime }}\right)}_{a_{n+1}a_{n+1}^{\prime },11}$. If k∈(2,…,n) and indices $i_{k}$ and $i_{k}^{\prime }$ are fixed, then we treat $M_{k}^{i_{k}i_{k}^{\prime }}$ as a matrix with elements ${\left(M_{k}^{i_{k}i_{k}^{\prime }}\right)}_{a_{k-1}a_{k-1}^{\prime },a_{k}a_{k}^{\prime }}$, i.e., $a_{k-1}a_{k-1}^{\prime }$ is a row multiindex and $a_{k}a_{k}^{\prime }$ is a column multiindex. The explicit expressions for *M*-tensors follow from Figure 6a,b and read

(10) $$\begin{array}{lll} & & {\left(M_{1}^{i_{1}i_{1}^{\prime }}\right)}_{11,a_{1}a_{1}^{\prime }}=\langle a_{1}|⊗\langle i_{1}|U\left(\varrho_{S}⊗\varrho_{1}\right)U^{†}|a_{1}^{\prime }\rangle ⊗|i_{1}^{\prime }\rangle , \end{array}$$

(11) $$\begin{array}{ccc} & & {\left(M_{k}^{i_{k}i_{k}^{\prime }}\right)}_{a_{k-1}a_{k-1}^{\prime },a_{k}a_{k}^{\prime }}=\langle a_{k}|⊗\langle i_{k}|U\left(|a_{k-1}\rangle \langle a_{k-1}^{\prime }|⊗\varrho_{k}\right)U^{†}|a_{k}^{\prime }\rangle ⊗|i_{k}^{\prime }\rangle ,\;k=2,\ldots ,n, \end{array}$$

(12) $$\begin{array}{ccc} & & {\left(M_{n+1}^{i_{n+1}i_{n+1}^{\prime }}\right)}_{a_{n+1}a_{n+1}^{\prime },11}=\delta_{i_{n+1},a_{n+1}}\delta_{i_{n+1}^{\prime },a_{n+1}^{\prime }}. \end{array}$$

Let $\varrho_{S}=\sum_{l}\lambda_{S}^{l}|\varphi_{S}^{l}\rangle \langle \varphi_{S}^{l}|$ be the spectral decomposition for the system initial state. Let $\varrho_{k}=\sum_{m}\lambda_{k}^{m}|\psi_{k}^{m}\rangle \langle \psi_{k}^{m}|$ be the spectral decomposition for the initial state of the *k*-th ancilla. Then one readily obtains the representation

(13) $$\begin{array}{ccc} & & M_{1}^{i_{1}i_{1}^{\prime }}=\sum_{lm}B_{lm}^{\left[1\right],i_{1}}⊗\bar{B_{lm}^{\left[1\right],i_{1}^{\prime }}},\;{\left(B_{lm}^{\left[1\right],i_{1}}\right)}_{1,a_{1}}=\sqrt{\lambda_{S}^{l}\lambda_{k}^{m}}\langle a_{1}|⊗\langle i_{1}|U|\varphi_{S}^{l}\rangle ⊗|\psi_{k}^{m}\rangle , \end{array}$$

(14) $$\begin{array}{ccc} & & M_{k}^{i_{k}i_{k}^{\prime }}=\sum_{m}B_{m}^{\left[k\right],i_{k}}⊗\bar{B_{m}^{\left[k\right],i_{k}^{\prime }}},\;{\left(B_{m}^{\left[k\right],i_{k}}\right)}_{a_{k-1},a_{k}}=\sqrt{\lambda_{k}^{m}}\langle a_{k}|⊗\langle i_{k}|U|a_{k-1}\rangle ⊗|\psi_{k}^{m}\rangle ,\;k=2,\ldots ,n,\; \end{array}$$

(15) $$\begin{array}{ccc} & & M_{n+1}^{i_{n+1}i_{n+1}^{\prime }}=B^{\left[n+1\right],i_{n+1}}⊗\bar{B^{\left[n+1\right],i_{n+1}}},\;{\left(B^{\left[n+1\right],i_{n+1}}\right)}_{a_{n},1}=\delta_{a_{n},i_{n+1}}. \end{array}$$

A tensor diagram for Equation (14) is depicted in Figure 6c. We see that for any k∈(1,2,…,n,n+1), the decomposition $M_{k}^{i_{k}i_{k}^{\prime }}=\sum_{b=1}^{D}B_{b}^{\left[k\right],i_{k}}⊗\bar{B_{b}^{\left[k\right],i_{k}^{\prime }}}$ takes place, with b=(lm) and $D\leq d_{S}d$ if k=1, b=m and D≤d if k∈(2,…,n), and b=D=1 if k=n+1. Tensor diagrams in Figure 6b,c define the so-called matrix product density operator (MPDO) [83,84], which is automatically Hermitian and positive semidefinite. MPDOs are successfully used to study the dissipative dynamics and the Gibbs states of one-dimensional quantum chains [83,84,85,86]. Among other questions, Ref. [86] addresses an important question how to prepare MPDO states experimentally. Our results show one more method to prepare an MPDO state via the standard collision model. In our construction, the MPDO is right canonical, i.e., it additionally satisfies the right-normalization condition

(16) $$\sum_{i_{k},a_{k}}{\left(M_{k}^{i_{k}i_{k}}\right)}_{a_{k-1}a_{k-1}^{\prime },a_{k}a_{k}}=\delta_{a_{k-1}a_{k-1}^{\prime }}\;\Leftrightarrow \;\sum_{i_{k},b}B_{b}^{\left[k\right],i_{k}}{\left(B_{b}^{\left[k\right],i_{k}}\right)}^{†}=I.$$

Equation (16) mathematically shows the independence of the reduced density operator $\varrho_{1\ldots k}\left(k\tau \right)$ for *k* ancillas from future collisions at times t>kτ. The results of this section are summarized as follows.

**Proposition** **3.** *The standard collision model with initially mixed states of the system ($\varrho_{S}$) and n ancillas ($\varrho_{1},\ldots ,\varrho_{n}$) produces a right-canonical MPDO (9) with elementary tensors given by Equations (10)–(12) and (13)–(15).*

The main benefit of the constructed MPDO representation is that it exploits only $d_{S}^{2}d^{2}+\left(n-1\right)d_{S}^{4}d^{2}\leq nd_{S}^{4}d^{2}$ parameters instead of $d_{S}^{2}d^{2n}$ parameters needed for a description of a general state of the system and *n* ancillas. In other words, computational resources scale linearly (not exponentially) with the number of ancillas if one uses the MPDO representation. This fact opens an avenue for further numerical studies in the collisional quantum thermometry [34,35]. If the system interacts with a thermal reservoir in between the collisions with ancillas, one can readily include such a system-reservoir interaction in the tensor network representation in the form of a quantum channel [87].

**Example** **2.** *Let the system and ancillas be qubits. The system is initially in the excited state |φ〉=|↑〉. Each ancilla is initially in the Gibbs state*

$$\varrho_{k}=\frac{1}{1+\exp[\left(E_{↓}-E_{↑}\right)/k_{B}T]}|↓\rangle \langle ↓|+\frac{1}{1+\exp[\left(E_{↑}-E_{↓}\right)/k_{B}T]}|↑\rangle \langle ↑|,$$

*where $k_{B}$ is the Boltzmann constant, T is the temperature, and $E_{↑}$ and $E_{↓}$ are the energy levels for the ancilla states |↑〉 and |↓〉, respectively. Consider the energy exchange unitary U=exp[gτ(|↓↑〉〈↑↓|−|↑↓〉〈↓↑|)]. After n collisions, the mixed state of the system and ancillas is fully described by a right-canonical MPDO with D=2. The explicit form for this MPDO is given by Proposition 3 and reads*

$$\begin{array}{ccc} & & B_{1}^{[1],↓}=\frac{1}{\sqrt{1+\exp[\left(E_{↓}-E_{↑}\right)/k_{B}T]}}\left(\begin{array}{cc} 0 & \cos g\tau \end{array}\right),\;B_{2}^{[1],↓}=\left(\begin{array}{cc} 0 & 0 \end{array}\right),\\ & & B_{1}^{[1],↑}=\frac{1}{\sqrt{1+\exp[\left(E_{↓}-E_{↑}\right)/k_{B}T]}}\left(\begin{array}{cc} \sin g\tau & 0 \end{array}\right),\;B_{2}^{[1],↑}=\frac{1}{\sqrt{1+\exp[\left(E_{↑}-E_{↓}\right)/k_{B}T]}}\left(\begin{array}{cc} 0 & 1 \end{array}\right),\\ & & B_{1}^{[k],↓}=\frac{1}{\sqrt{1+\exp[\left(E_{↓}-E_{↑}\right)/k_{B}T]}}\left(\begin{array}{cc} 1 & 0\\ 0 & \cos g\tau \end{array}\right),\;B_{2}^{[k],↓}=\frac{1}{\sqrt{1+\exp[\left(E_{↑}-E_{↓}\right)/k_{B}T]}}\left(\begin{array}{cc} 0 & -\sin g\tau \\ 0 & 0 \end{array}\right),\\ & & B_{1}^{[k],↑}=\frac{1}{\sqrt{1+\exp[\left(E_{↓}-E_{↑}\right)/k_{B}T]}}\left(\begin{array}{cc} 0 & 0\\ \sin g\tau & 0 \end{array}\right),\;B_{2}^{[k],↑}=\frac{1}{\sqrt{1+\exp[\left(E_{↑}-E_{↓}\right)/k_{B}T]}}\left(\begin{array}{cc} \cos g\tau & 0\\ 0 & 1 \end{array}\right),\\ & & B^{[n+1],↓}=\left(\begin{array}{c} 1\\ 0 \end{array}\right)\;B^{[n+1],↑}=\left(\begin{array}{c} 0\\ 1 \end{array}\right), \end{array}$$

*where k=2,…,n.*

## 5. Collision Model with a Generally Correlated State of Ancillas

Let us consider a more complicated collision model, in which ancillas are initially correlated. Surprisingly enough, any pure state of *n* ancillas adopts an MPS representation [73,74,75,76,77,78]. However, the MPS rank for a generally correlated state grows exponentially with *n*. On the other hand, many important states of correlated ancillas such as few-photon wavepackets [46,47,48,49], artificial photonic tensor network states [37,88,89,90,91,92,93], and ground states of gapped one-dimensional local Hamiltonians for the spin chains [94] are described by MPSs with a low MPS rank. As the states of ancillas are mixed in general, we exploit the MPDO formalism. We pay little attention to the rank of decomposition as our further goal is to reveal the effect of ancillas’ correlations on the system dynamics. Note that the correlations can be either quantum (genuinely entangled ancillas) or classical (fully separable state of ancillas); however, both types strongly affect the system dynamics (see an example in Ref. [50]).

**Figure 7:**
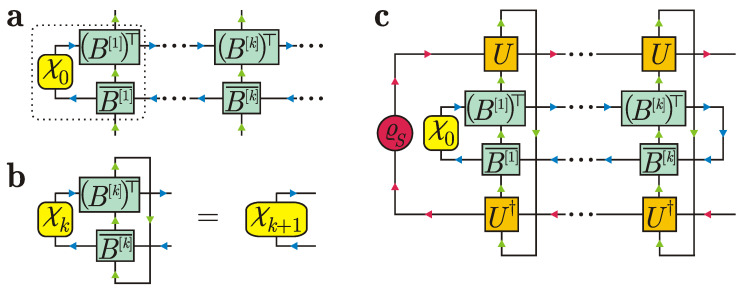
(**a**) Matrix product density operator representation for a generally correlated state of ancillas, where we have formally added an auxiliary density matrix $\chi_{0}$ for the bond degrees of freedom to redefine the first MPDO tensor (dotted region). (**b**) Recurrence relation of density operators for bond degrees of freedom in the interaction-free evolution. (**c**) Tensor diagram for the system density operator $\varrho_{S}\left(k\tau \right)$ after *k* collisions with correlated ancillas.

Let the intial state $\varrho_{1\ldots n}$ be a right-canonical MPDO for *n* ancillas shown in Figure 7a. Here, we have added a formal density operator $\chi_{0}$ (i.e., a positive semidefinite operator with unit trace) for the bond degrees of freedom (blue arrows in Figure 7a). In the conventional MPDO notation, $\chi_{0}$ is the trivial 1×1 identity matrix for dummy indices; however, in our construction, it can be an arbitrary density matrix such that the tensor contraction is well defined. Note that we changed the direction of arrows in the upper line in Figure 7a. This implies transposition of matrices $B_{b}^{\left[k\right],i_{k}}$ with respect to horizontal virtual indices.

Figure 7b illustrates that the tensors ${\left\{B^{\left[k\right]}\right\}}_{k}$ define a “free evolution” for the bond degrees of freedom if ancillas do not interact with the system; namely, the matrix

(17) $$\chi_{k+1}=\sum_{i_{k},b}{\left(B_{b}^{\left[k\right],i_{k}}\right)}^{⊤}\chi_{k}\bar{B_{b}^{\left[k\right],i_{k}}}$$

is a valid density matrix (i.e., $\chi_{k+1}^{†}=\chi_{k+1}\geq 0$ and $\mathrm{tr}[\chi_{k}]=1$) provided $\chi_{k}$ is also a density matrix. This follows from the right normalization condition (16).

Figure 7c depicts the system density operator $\varrho_{S}\left(k\tau \right)$ after *k* collisions. The partial trace over ancillas k+1,…,n corresponds to a vertical connecting line for the bond degrees of freedom (blue arrows in Figure 7c). The partial trace over ancillas 1,…,k corresponds to vertical connecting lines for the ancillary degrees of freedom (green arrows in Figure 7c). Ref. [72] discusses the natural Markovian embedding for the system dynamics that follows from the diagram in Figure 7c. In our case, we have

(18) $$\varrho_{S}\left(k\tau \right)={\mathrm{tr}}_{\mathrm{bond}}\left[E^{\left[k\right]}\circ \ldots \circ E^{\left[1\right]}\left[\varrho_{S}⊗\chi_{0}\right]\right],$$

where ∘ denotes a map concatenation. Each map $E^{\left[m\right]}$ is completely positive and trace preserving because it adopts the diagonal sum representation

(19) $$E^{\left[m\right]}\left[R\right]=\sum_{j_{m}b}K_{j_{m}b}\;R\;K_{j_{m}b}^{†},\;K_{j_{m}b}=\sum_{i_{m}}\left(\left(I_{S}⊗\langle j_{m}|\right)U\left(I_{S}⊗|i_{m}\rangle \right)\right)⊗{\left(B_{b}^{\left[m\right],i_{m}}\right)}^{⊤}.$$

The trace preserving property $\sum_{j_{m}b}K_{j_{m}b}^{†}K_{j_{m}b}=I$ follows from the right normalization condition (16) and unitarity of *U*.

The tensor diagram in Figure 7c is a particular form of the process tensor—a recently developed approach to an operational description of non-Markovian quantum dynamics [59,95,96,97,98]. The complexity of the non-Markovian dynamics simulation depends on the dimension of the effective reservoir in the Markovian embedding [99,100]: the less the dimension of the Markovian embedding, the simpler the simulation. In our model, the role of the effective reservoir is played by the bond degrees of freedom that specify correlations among the ancillas.

The emergence of non-Markovian dynamics in the case of correlated ancillas was demonstrated in Ref. [50], where an exemplary indecomposable qubit channel was realized as a result of a qubit’s collisional interactions with many qutrit ancillas in the GHZ state. The analytical treatment in Ref. [50] was only possible due to a peculiar controlled-unitary qubit-ancilla interaction. Were the qubit-ancilla interaction different from the controlled-unitary type, the methods of Ref. [50] would not provide any analytical expression for the qubit system dynamics (nor would it be possible to study its non-Markovianity). As we show in the example below, the developed tensor network formalism enables us to resolve that difficulty and analytically derive the qubit dynamics even for non-controlled unitary collisions. Since any environment state adopts an MPDO form, our results generalize those of Ref. [51], where non-Markovian qubit dynamics are induced by a specific correlated environment $\varrho_{1\ldots n}={⊕}_{m}p_{m}{⊗}_{k=1}^{n}\varrho_{k}^{\left(m\right)}$ or $\varrho_{1\ldots n}={⊗}_{m=x,y,z}\left(\frac{1}{2}{⊗}_{k\in \{k_{m}\}}|i_{k}\rangle \langle i_{k}|+\frac{1}{2}{⊗}_{k\in \{k_{m}\}}|\bar{i_{k}}\rangle \langle \bar{i_{k}}|\right)$, where either $i_{k}=0$ and $\bar{i_{k}}=1$, or $i_{k}=1$ and $\bar{i_{k}}=0$; $\left\{k_{x}\right\}$, $\left\{k_{y}\right\}$, $\left\{k_{z}\right\}$ are nonintersecting subsequences of collision numbers. The latter environment reproduces an arbitrary Pauli dynamical map [51].

**Example** **3.** *Consider the GHZ state of 3-dimensional ancillas $\varrho_{1\ldots n}=|\mathrm{GHZ}\rangle \langle \mathrm{GHZ}|$, $|\mathrm{GHZ}\rangle =\frac{1}{\sqrt{3}}\sum_{j=1,2,3}{|j\rangle }^{⊗n}$. The tensor network representation for this pure state reads*

(20) $$\chi_{0}=\frac{1}{3}\left(\begin{array}{ccc} 1 & 1 & 1\\ 1 & 1 & 1\\ 1 & 1 & 1 \end{array}\right),\;B_{1}^{[k],1}=\left(\begin{array}{ccc} 1 & 0 & 0\\ 0 & 0 & 0\\ 0 & 0 & 0 \end{array}\right),\;B_{1}^{[k],2}=\left(\begin{array}{ccc} 0 & 0 & 0\\ 0 & 1 & 0\\ 0 & 0 & 0 \end{array}\right),\;B_{1}^{[k],3}=\left(\begin{array}{ccc} 0 & 0 & 0\\ 0 & 0 & 0\\ 0 & 0 & 1 \end{array}\right).$$

*The qubit system is initially in the state $\varrho_{S}\equiv \varrho_{S}\left(0\right)$. Each qubit-qutrit collision is described by the unitary operator*

(21) $$U=\exp\left[-i\frac{g\tau }{2}\sum_{j=1,2,3}\sigma_{j}⊗J_{j}\right],$$

*where $\left(\sigma_{1},\sigma_{2},\sigma_{3}\right)\equiv \left(\sigma_{x},\sigma_{y},\sigma_{z}\right)$ is the conventional set of Pauli operators, and $\left(J_{1},J_{2},J_{3}\right)\equiv \left(J_{x},J_{y},J_{z}\right)$ is a set of SU(2) generators for a qutrit (spin-1 particle). In the conventional orthonormal basis (|1〉,|2〉,|3〉), the corresponding matrices are*

(22) $$J_{x}=\frac{1}{\sqrt{2}}\left(\begin{array}{ccc} 0 & 1 & 0\\ 1 & 0 & 1\\ 0 & 1 & 0 \end{array}\right),\;J_{y}=\frac{1}{\sqrt{2}}\left(\begin{array}{ccc} 0 & -i & 0\\ i & 0 & -i\\ 0 & i & 0 \end{array}\right),\;J_{z}=\left(\begin{array}{ccc} 1 & 0 & 0\\ 0 & 0 & 0\\ 0 & 0 & -1 \end{array}\right).$$

*Substituting Equations (20) and (21) into Equation (19), we get the map $E^{\left[m\right]}\equiv E$, which does not depend on the collision number m. Then Equation (18) results in the following qubit system density operator after k collisions:*

(23) $$\varrho_{S}\left(k\tau \right)=\frac{1}{2}\left\{I+\lambda \left(k\right)\mathrm{tr}\left[\varrho_{S}\left(0\right)\sigma_{x}\right]\sigma_{x}+\lambda \left(k\right)\mathrm{tr}\left[\varrho_{S}\left(0\right)\sigma_{y}\right]\sigma_{y}+\lambda_{z}\left(k\right)\mathrm{tr}\left[\varrho_{S}\left(0\right)\sigma_{z}\right]\sigma_{z}\right\},$$

*where λ(k) is a scaling coefficient for the x and y components of the qubit Bloch vector, and $\lambda_{z}\left(k\right)$ is a scaling coefficient for the z component of the qubit Bloch vector. The center of the Bloch ball is a steady point under such dynamics. The explicit formulas for the scaling coefficients are*

(24) $$\begin{array}{ccc} \lambda (k) & = & \frac{3^{k}{\left[1+2\exp\left(3ig\tau /2\right)\right]}^{k}+3^{k}{\left[1+2\exp\left(-3ig\tau /2\right)\right]}^{k}+{\left[5+4\cos\left(3g\tau /2\right)\right]}^{k}}{3^{2k+1}}\;,\; \end{array}$$

(25) $$\begin{array}{ccc} \lambda_{z}\left(k\right) & = & \frac{{\left[1+8\cos\left(3g\tau /2\right)\right]}^{k}+2{\left[5+4\cos\left(3g\tau /2\right)\right]}^{k}}{3^{2k+1}}\;. \end{array}$$

**Figure 8:**
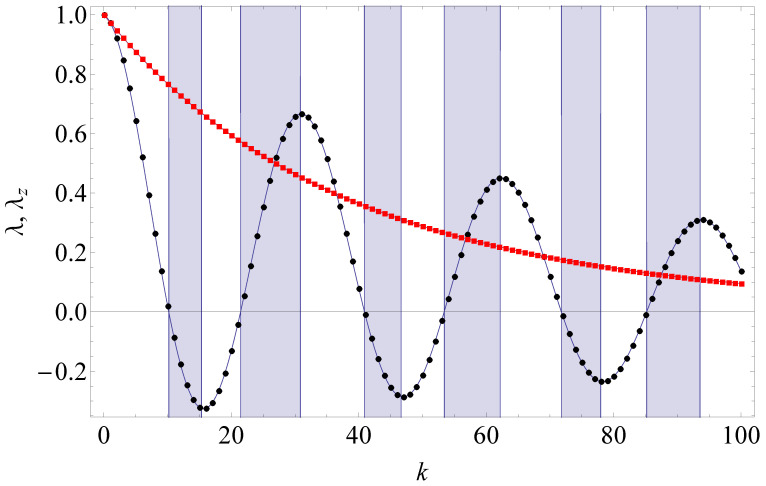
Dephasing coefficient λ (black circles) and amplitude relaxation coefficient $\lambda_{z}$ (red squares) vs number of collisions *k* in qubit dynamics (23) with gτ=0.2 emerging in the collision model with the correlated GHZ state of qutrit ancillas. Colored regions correspond to essential non-Markovianity (positive indivisibility) of the qubit dynamics.

*We portray the typical behaviour of λ(k) and $\lambda_{z}\left(k\right)$ in Figure 8. Whenever |λ(k)| increases with increasing k, we observe a positive indivisible dynamic, which is often treated as an indication of essential non-Markovianity [101]. We refer the interested reader to Ref. [102] for a full analysis of divisibility properties under the phase covariant qubit dynamics — the class of quantum dynamical maps comprising the map (23).*

## 6. Master Equation

Equation (18) defines the discrete dynamical map $\Upsilon_{k\tau }$ that transforms the initial system density operator $\varrho_{S}\left(0\right)\equiv \varrho_{S}$ to the system density operator $\varrho_{S}\left(k\tau \right)$ after *k* collisions, i.e., $\Upsilon_{k\tau }\left[\varrho_{S}\left(0\right)\right]=\varrho_{S}\left(k\tau \right)$. If τ→0, then we interpret kτ as a continuous time *t*. A time-local master equation $\frac{d\varrho_{S}\left(t\right)}{dt}=L_{t}\left[\varrho_{S}\left(t\right)\right]$ can be derived if $\Upsilon_{t}$ is invertible; namely, $L_{t}=\frac{d\Upsilon_{t}}{dt}\circ \Upsilon_{t}^{-1}$. Although such a master equation correctly describes the system evolution, it conceals the major role of correlations among ancillas. To reveal the physics of how these correlations affect the system dynamics, we resort to the conventional projection operator techniques [103] and derive the Nakajima–Zwanzig memory kernel equation [104,105] for our model.

Let ${\left\{\chi_{k}\right\}}_{k}$ be a collection of the density operators for the bond degrees of freedom generated by Equation (17). For each *k*, define the following map $P_{k}$ acting on both the system and the bond degrees of freedom:

(26) $$P_{k}\left[R\right]={\mathrm{tr}}_{\mathrm{bond}}\left[R\right]⊗\chi_{k}.$$

**Figure 9:**
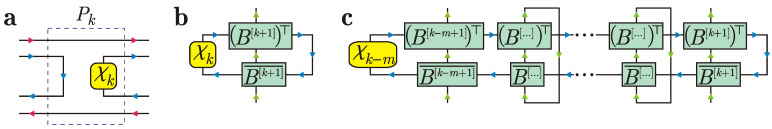
(**a**) Projection $P_{k}$. (**b**) Reduced density operator $\varrho_{k+1}$ for the (k+1)-th ancilla in the initial correlated state of ancillas. (**c**) Reduced density operator $\varrho_{k-m+1,k+1}$ for the (k−m+1)-th ancilla and (k+1)-th ancilla in the initial correlated state of ancillas.

Since $\mathrm{tr}[\chi_{k}]=1$, we have $P_{k}^{2}=P_{k}$, so $P_{k}$ is a projection. A pictorial representation of the projection $P_{k}$ is given in Figure 9a, which shows that $P_{k}$ breaks the left-right correlations between bunches of ancillas (1,…,k) and (k+1,…,n). Clearly,

(27) $$P_{k}\left[E^{\left[k\right]}\circ \ldots \circ E^{\left[1\right]}\left[\varrho_{S}\left(0\right)⊗\chi_{0}\right]\right]=\varrho_{S}\left(k\tau \right)⊗\chi_{k}.$$

A complementary projection $Q_{k}$ is defined through

(28) $$Q_{k}={\mathrm{Id}}_{S+\mathrm{bond}}-P_{k},$$

where Id is the identity transformation for the system and bond degrees of freedom. $Q_{k}$ is also a projection because $Q_{k}^{2}=Q_{k}$. We can also rewrite $Q_{k}$ in the form

(29) $$Q_{k}={\mathrm{Id}}_{S}⊗Q_{\mathrm{bond}\#k},$$

where $Q_{\mathrm{bond}\#k}$ is a projection for the *k*-th bond degrees of freedom that acts on an operator *F* for the bond degrees of freedom as follows:

(30) $$Q_{\mathrm{bond}\#k}\left[F\right]=F-\mathrm{tr}\left[F\right]\chi_{k}.$$

Since $Q_{\mathrm{bond}\#0}\left[\chi_{0}\right]=0$, we readily get

(31) $$Q_{0}\left[\varrho_{S}\left(0\right)⊗\chi_{0}\right]=0.$$

To simplify the notation, let us introduce the system-bond density operator after the *k*-th collision, $R\left(k\tau \right)=E^{\left[k\right]}\circ \ldots \circ E^{\left[1\right]}\left[\varrho_{S}\left(0\right)⊗\chi_{0}\right]$. Then, $\left[R\left(k\tau \right)\right]=E^{\left[k\right]}\left[R\left((k-1)\tau \right)\right]$ for all *k*. Applying $Q_{k}$ to the both sides of the latter equation, we get

(32) $$Q_{k}\left[R\left(k\tau \right)\right]=Q_{k}\circ E^{\left[k\right]}\circ \left(P_{k-1}+Q_{k-1}\right)\left[R\left((k-1)\tau \right)\right].$$

The recurrent Equation (32) with the initial condition (31) has the following formal solution:

(33) $$Q_{k}\left[R\left(k\tau \right)\right]=\sum_{m=1}^{k}Q_{k}\circ E^{\left[k\right]}\circ \ldots \circ Q_{k-m+1}\circ E^{\left[k-m+1\right]}\circ P_{k-m}\left[R\left((k-m)\tau \right)\right].$$

If we apply $P_{k+1}$ to the both sides of equation $[R\left(\left(k+1\right)\tau )\right]=E^{\left[k+1\right]}\left[R(k\tau )\right]$, we obtain

(34) $$\begin{array}{ccc} & & P_{k+1}\left[R\left((k+1)\tau \right)\right]=P_{k+1}\circ E^{\left[k+1\right]}\circ \left(P_{k}+Q_{k}\right)\left[R(k\tau )\right]=P_{k+1}\circ E^{\left[k+1\right]}\circ P_{k}\left[R(k\tau )\right]+P_{k+1}\circ E^{\left[k+1\right]}Q_{k}\left[R(k\tau )\right]\\ & & =P_{k+1}\circ E^{\left[k+1\right]}\circ P_{k}\left[R(k\tau )\right]+\sum_{m=1}^{k}P_{k+1}\circ E^{\left[k+1\right]}\circ Q_{k}\circ E^{\left[k\right]}\circ \ldots \circ Q_{k-m+1}\circ E^{\left[k-m+1\right]}\circ P_{k-m}\left[R\left((k-m)\tau \right)\right]. \end{array}$$

Recalling the relation $P_{k}\left[R(k\tau )\right]=\varrho_{S}\left(k\tau \right)⊗\chi_{k}$ and taking partial trace over the bond indices in Equation (34), we get

(35) $$\begin{array}{c} \varrho_{S}\left((k+1)\tau \right)={\mathrm{tr}}_{\mathrm{bond}}\circ E^{\left[k+1\right]}\left[\varrho_{S}\left(k\tau \right)⊗\chi_{k}\right]+\sum_{m=1}^{k}{\mathrm{tr}}_{\mathrm{bond}}\circ E^{\left[k+1\right]}\circ Q_{k}\circ E^{\left[k\right]}\circ \ldots \circ Q_{k-m+1}\circ E^{\left[k-m+1\right]}\\ \left[\varrho_{S}\left((k-m)\tau \right)⊗\chi_{k-m}\right]. \end{array}$$

Subtracting $\varrho_{S}\left(k\tau \right)$ from both sides of Equation (35) and dividing the result by the collision time τ, we get a discrete-time version of the celebrated Nakajima–Zwanzig master equation, namely,

(36) $$\frac{\varrho_{S}\left((k+1)\tau \right)-\varrho_{S}\left(k\tau \right)}{\tau }=\sum_{m=0}^{k}K_{km}\left[\varrho_{S}\left((k-m)\tau \right)\right],$$

where the memory kernel $K_{km}$ relates the density matrix increment (in between the times kτ and (k+1)τ) with the past density operator at time (k−m)τ. If m=0, then we have a time-local term $K_{k0}$ giving the density operator increment caused by the latest collision (among those that have already happened):

(37) $$K_{k0}\left[\varrho_{S}\right]=\frac{{\mathrm{tr}}_{k+1}\left[U\varrho_{S}⊗\varrho_{k+1}U^{†}\right]-\varrho_{S}}{\tau },$$

with $\varrho_{k+1}$ being a reduced density operator for the (k+1)-th ancilla in the initial state; see Figure 9b. If m≥1, then $K_{km}$ describes a nontrivial effect of preceding collisions on the system evolution and reads

(38) $$K_{km}\left[\varrho_{S}\right]=\frac{1}{\tau }{\mathrm{tr}}_{\mathrm{bond}}\circ E^{\left[k+1\right]}\circ Q_{k}\circ E^{\left[k\right]}\circ \ldots \circ Q_{k-m+1}\circ E^{\left[k-m+1\right]}\left[\varrho_{S}⊗\chi_{k-m}\right].$$

If there were no correlations in the environment, then $K_{k0}$ would be the only contribution to the kernel because $K_{km}$ would vanish for all m≥1. Indeed, the MPDO rank equals 1 for a factorized environment state, so $\dim H_{\mathrm{bond}\#k}=1$ for all *k*. Each $\chi_{k}$ is unambiguously defined because $\chi_{k}$ is the trivial 1×1 identity matrix in this case, and $Q_{\mathrm{bond}\#k}\left[F\right]=0$ for any 1×1 matrix *F*. If the environment is correlated, then the memory contribution $K_{km}\left[\varrho_{S}\right]\neq 0$ in general.

**Example** **4.** *Consider the GHZ state of 3-dimensional ancillas $\varrho_{1\ldots n}=|\mathrm{GHZ}\rangle \langle \mathrm{GHZ}|$, $|\mathrm{GHZ}\rangle =\frac{1}{\sqrt{3}}\sum_{j=1,2,3}{|j\rangle }^{⊗n}$ and the controlled unitary system-ancilla interaction $U=\sum_{j=1,2,3}e^{-ig\tau \sigma_{j}}⊗|j\rangle \langle j|$, where gτ quantifies the dimensionless system-ancilla interaction strength and $\left(\sigma_{1},\sigma_{2},\sigma_{3}\right)\equiv \left(\sigma_{x},\sigma_{y},\sigma_{z}\right)$ is the conventional set of Pauli operators. This is a scenario considered also in Ref. [50]. A direct calculation yields*

(39) $$K_{km}\left[\varrho_{S}\right]=\frac{1}{3\tau }\sum_{i_{k+1},\ldots ,i_{k-m+1}=1}^{3}\left[\prod_{l=k-m+1}^{k}\left(\delta_{i_{l},i_{l+1}}-\frac{1}{3}\right)\right]e^{-ig\tau \sigma_{i_{k+1}}}\cdots e^{-ig\tau \sigma_{i_{k-m+1}}}\varrho_{S}e^{ig\tau \sigma_{i_{k-m+1}}}\cdots e^{ig\tau \sigma_{i_{k+1}}}.$$

*The memory kernel $K_{km}$ does not decay with the increase of m due to the infinite correlation length in the GHZ state. In view of this, even if the interaction strength gτ≪1, one cannot truncate a series expansion for $K_{km}$ with respect to a small parameter gτ. Instead, all orders of gτ are significant for reproducing the system dynamics.*

If the correlation length is finite, then it is possible to derive a continuous-time master equation in the appropriate limit for τ and *g*. This is discussed in what follows.

## 7. Effect of Two-Point Correlations

An important simplification comes from a series expansion for $U_{Sm}$ with respect to the interaction strength gτ between the system and an individual environment particle. Let $g\hbar H_{m}$ be the system-particle interaction Hamiltonian during the *m*-th collision, where *ℏ* is the reduced Planck constant, *g* has the physical dimension of frequency, and $H_{m}$ is a dimensionless Hermitian operator with the operator norm $∥H_{m}∥\leq 1$. Then, the elementary unitary interaction in the *m*-th collision is $U=\exp(-ig\tau H_{m})$. The map $E^{\left[m\right]}$ in Equation (19) has a contribution of both *U* and $U^{†}$, so we have

(40) $$\begin{array}{ccc} & & E^{\left[k\right]}=\sum_{i_{k},i_{k}^{\prime }}\Phi_{i_{k}i_{k}^{\prime }}^{\left[k\right]}⊗\Lambda_{i_{k}i_{k}^{\prime }}^{\left[k\right]},\\ & & \Phi_{i_{k}i_{k}^{\prime }}^{\left[k\right]}\left[\varrho_{S}\right]\equiv {\mathrm{tr}}_{k}\left[U\;\varrho_{S}⊗|i_{k}\rangle \langle i_{k}^{\prime }|\;U\right]\\ & & \;=\delta_{i_{k}i_{k}^{\prime }}\varrho_{S}-ig\tau \left[\langle i_{k}^{\prime }|H_{k}|i_{k}\rangle ,\varrho_{S}\right]+g^{2}\tau^{2}\left(\sum_{j_{k}=1}^{d}\langle j_{k}|H_{k}|i_{k}\rangle \varrho_{S}\langle i_{k}^{\prime }|H_{k}|j_{k}\rangle -\frac{1}{2}\left\{\langle i_{k}^{\prime }|H_{k}^{2}|i_{k}\rangle ,\varrho_{S}\right\}\right)+o\left(g^{2}\tau^{2}\right) \end{array}$$

(41) $$\begin{array}{ccc} & & \;\equiv \Phi_{i_{k}i_{k}^{\prime }}^{[k],\left(0\right)}\left[\varrho_{S}\right]+g\tau \Phi_{i_{k}i_{k}^{\prime }}^{[k],\left(1\right)}\left[\varrho_{S}\right]+g^{2}\tau^{2}\Phi_{i_{k}i_{k}^{\prime }}^{[k],\left(2\right)}\left[\varrho_{S}\right]+o\left(g^{2}\tau^{2}\right), \end{array}$$

(42) $$\begin{array}{ccc} & & \Lambda_{i_{k}i_{k}^{\prime }}^{\left[k\right]}\left[•\right]=\sum_{b}{\left(B_{b}^{\left[k\right],i_{k}^{\prime }}\right)}^{⊤}•\bar{B_{b}^{\left[k\right],i_{k}}}, \end{array}$$

where [•,•] and {•,•} denote the commutator and the anticommutator, respectively. Substituting Equation (41) into Equation (38), we keep track of the leading terms in the memory kernel, namely,

(43) $$K_{km}=\frac{1}{\tau }K_{km}^{\left(0\right)}+gK_{km}^{\left(1\right)}+g^{2}\tau K_{km}^{\left(2\right)}+o\left(g^{2}\tau \right).$$

The term $K_{km}^{\left(0\right)}$ vanishes because

(44) $$Q_{\mathrm{bond}\#(k-m+1)}\circ \sum_{i_{k-m+1}}\Lambda_{i_{k-m+1}i_{k-m+1}}^{\left[k-m+1\right]}\left[\chi_{k-m}\right]=Q_{\mathrm{bond}\#(k-m+1)}\left[\chi_{k-m+1}\right]=0;$$

see Equation (30). Physically, the 0-th order of $\Phi_{i_{k},i_{k}^{\prime }}^{\left[k\right]}$ involves no system-environment interaction and, consequently, no contribution to the memory kernel.

To calculate the term $K_{km}^{\left(1\right)}$, we should fix $\Phi_{i_{l},i_{l}^{\prime }}^{\left[l\right]}=\delta_{i_{l}i_{l}^{\prime }}{\mathrm{Id}}_{S}\equiv \Phi_{i_{l},i_{l}^{\prime }}^{\left[l],(0\right)}$ for all but one of l∈(k−m+1,…,k+1). If $\Phi_{i_{k-m+1},i_{k-m+1}^{\prime }}^{\left[k-m+1\right]}=\delta_{i_{k-m+1}i_{k-m+1}^{\prime }}{\mathrm{Id}}_{S}$, then we have a zero contribution to $K_{km}^{\left(1\right)}$ because of Equation (44). Suppose $\Phi_{i_{k-m+1},i_{k-m+1}^{\prime }}^{\left[k-m+1\right]}\neq \delta_{i_{k-m+1}i_{k-m+1}^{\prime }}{\mathrm{Id}}_{S}$; then, $\Phi_{i_{k+1},i_{k+1}^{\prime }}^{\left[k+1\right]}=\delta_{i_{k+1}i_{k+1}^{\prime }}{\mathrm{Id}}_{S}$, and

(45) $$\mathrm{tr}[\sum_{i_{k+1}}\Lambda_{i_{k+1}i_{k+1}}^{\left[k+1\right]}\circ Q_{\mathrm{bond}\#k}\left[•\right]]=\mathrm{tr}[Q_{\mathrm{bond}\#k}\left[•\right]]=0,$$

because $\sum_{i_{k+1}}\Lambda_{i_{k+1}i_{k+1}}^{\left[k+1\right]}$ is a trace preserving map due to the right-normalization condition, whereas $Q_{\mathrm{bond}\#k}$ nullifies the trace of any operator (see Equation (30)). Therefore, the term $K_{km}^{\left(1\right)}$ also vanishes.

Similar considerations for the term $K_{km}^{\left(2\right)}$ lead to a conclusion that $K_{km}^{\left(2\right)}$ may be nonzero only if we fix $\Phi_{i_{l},i_{l}^{\prime }}^{\left[l\right]}=-ig\tau \left[\langle i_{l}^{\prime }|H_{l}|i_{l}\rangle ,\varrho_{S}\right]\equiv g\tau \Phi_{i_{l},i_{l}^{\prime }}^{\left[l],(1\right)}$ for l=k+1 and l=k−m+1, whereas for all l=k−m+2,…,k we fix $\Phi_{i_{l},i_{l}^{\prime }}^{\left[l\right]}=\delta_{i_{l}i_{l}^{\prime }}{\mathrm{Id}}_{S}\equiv \Phi_{i_{l},i_{l}^{\prime }}^{\left[l],(0\right)}$. This results in

(46) $$\begin{array}{ccc} K_{km}^{\left(2\right)}\left[\varrho_{S}\right] & = & \sum_{i_{k+1},i_{k+1}^{\prime },i_{k-m+1},i_{k-m+1}^{\prime }}C_{i_{k-m+1}i_{k-m+1}^{\prime }i_{k+1}i_{k+1}^{\prime }}^{\left(2\right)}\Phi_{i_{k+1}i_{k+1}^{\prime }}^{[k+1],\left(1\right)}\circ \Phi_{i_{k-m+1}i_{k-m+1}^{\prime }}^{[k-m+1],\left(1\right)}\left[\varrho_{S}\right]\\ & = & -\sum_{i_{k+1},i_{k+1}^{\prime },i_{k-m+1},i_{k-m+1}^{\prime }}C_{i_{k-m+1}i_{k-m+1}^{\prime }i_{k+1}i_{k+1}^{\prime }}^{\left(2\right)}\left[\langle i_{k+1}^{\prime }|H_{k+1}|i_{k+1}\rangle ,\left[\langle i_{k-m+1}^{\prime }|H_{k-m+1}|i_{k-m+1}\rangle ,\varrho_{S}\right]\right], \end{array}$$

where the coefficient $C_{i_{k-m+1}i_{k-m+1}^{\prime }i_{k+1}i_{k+1}^{\prime }}^{\left(2\right)}$ reads

(47) $$\begin{array}{ccc} & & C_{i_{k-m+1}i_{k-m+1}^{\prime }i_{k+1}i_{k+1}^{\prime }}^{\left(2\right)}\\ & & =\mathrm{tr}[\Lambda_{i_{k+1}i_{k+1}^{\prime }}^{\left[k+1\right]}\circ Q_{\mathrm{bond}\#k}\circ \sum_{i_{k}}\Lambda_{i_{k}i_{k}}^{\left[k\right]}\circ \ldots \circ Q_{\mathrm{bond}\#(k-m+2)}\circ \sum_{i_{k-m+2}}\Lambda_{i_{k-m+2}i_{k-m+2}}^{\left[k-m+2\right]}\circ \\ & & Q_{\mathrm{bond}\#(k-m+1)}\circ \Lambda_{i_{k-m+1}i_{k-m+1}^{\prime }}^{\left[k-m+1\right]}\left[\chi_{k-m}\right]]. \end{array}$$

Recalling the definition (30), we get

(48) $$\begin{array}{c} Q_{\mathrm{bond}\#k}\circ \sum_{i_{k}}\Lambda_{i_{k}i_{k}}^{\left[k\right]}\circ \ldots \circ Q_{\mathrm{bond}\#(k-m+2)}\circ \sum_{i_{k-m+2}}\Lambda_{i_{k-m+2}i_{k-m+2}}^{\left[k-m+2\right]}\circ \\ Q_{\mathrm{bond}\#(k-m+1)}=\sum_{i_{k},\ldots ,i_{k-m+2}}\Lambda_{i_{k}i_{k}}^{\left[k\right]}\circ \ldots \circ \Lambda_{i_{k-m+2}i_{k-m+2}}^{\left[k-m+2\right]}-P_{k}. \end{array}$$

Tensor representation in Figure 9c justifies that

(49) $$\begin{array}{c} \mathrm{tr}[\Lambda_{i_{k+1}i_{k+1}^{\prime }}^{\left[k+1\right]}\circ \sum_{i_{k},\ldots ,i_{k-m+2}}\Lambda_{i_{k}i_{k}}^{\left[k\right]}\circ \ldots \circ \Lambda_{i_{k-m+2}i_{k-m+2}}^{\left[k-m+2\right]}\circ \Lambda_{i_{k-m+1}i_{k-m+1}^{\prime }}^{\left[k-m+1\right]}\left[\chi_{k-m}\right]]=\varrho_{k-m+1,k+1}, \end{array}$$

i.e., we get the reduced density operator for the (k−m+1)-th ancilla and (k+1)-th ancilla in the initial correlated state of ancillas. Similarly,

(50) $$\begin{array}{c} \mathrm{tr}\left[\Lambda_{i_{k+1}i_{k+1}^{\prime }}^{\left[k+1\right]}\circ P_{k}\circ \Lambda_{i_{k-m+1}i_{k-m+1}^{\prime }}^{\left[k-m+1\right]}\left[\chi_{k-m}\right]\right]=\varrho_{k-m+1}⊗\varrho_{k+1}, \end{array}$$

i.e., we get a tensor product of individual reduced density operators for the (k−m+1)-th ancilla and (k+1)-th ancilla in the initial correlated state of ancillas.

Combining (47)–(50), we obtain a surprisingly simple though exact result, namely,

(51) $$C_{i_{k-m+1}i_{k-m+1}^{\prime }i_{k+1}i_{k+1}^{\prime }}^{\left(2\right)}=\langle i_{k-m+1}i_{k+1}|\varrho_{k-m+1,k+1}|i_{k-m+1}^{\prime }i_{k+1}^{\prime }\rangle -\langle i_{k-m+1}|\varrho_{k-m+1}|i_{k-m+1}^{\prime }\rangle \langle i_{k+1}|\varrho_{k+1}|i_{k+1}^{\prime }\rangle .$$

Introducing the environment two-point correlation function for operators *O* and $O^{\prime }$ by a conventional formula

(52) $$\begin{array}{ccc} C(O,O^{\prime }) & = & {\langle OO^{\prime }\rangle }_{\mathrm{anc}}-{\langle O\rangle }_{\mathrm{anc}}{\langle O^{\prime }\rangle }_{\mathrm{anc}} \end{array}$$

(53) $$\begin{array}{ccc} & = & \mathrm{tr}\left[O⊗O^{\prime }\left(\varrho_{k-m+1,k+1}-\varrho_{k-m+1}⊗\varrho_{k+1}\right)\right], \end{array}$$

we readily see that $C_{i_{k-m+1}i_{k-m+1}^{\prime }i_{k+1}i_{k+1}^{\prime }}^{\left(2\right)}=C\left(O,O^{\prime }\right)$, where $O=|i_{k-m+1}^{\prime }\rangle \langle i_{k-m+1}|$ and $O^{\prime }=|i_{k+1}^{\prime }\rangle \langle i_{k+1}|$. Combining all the findings of this section, we get

(54) $$\begin{array}{ccc} K_{km}\left[\varrho_{S}\right] & = & -g^{2}\tau \sum_{i_{k+1},i_{k+1}^{\prime },i_{k-m+1},i_{k-m+1}^{\prime }}C\left(|i_{k-m+1}^{\prime }\rangle \langle i_{k-m+1}|,|i_{k+1}^{\prime }\rangle \langle i_{k+1}|\right)\\ & & \;\;\times \left[\langle i_{k+1}^{\prime }|H_{k+1}|i_{k+1}\rangle ,\left[\langle i_{k-m+1}^{\prime }|H_{k-m+1}|i_{k-m+1}\rangle ,\varrho_{S}\right]\right]+o\left(g^{2}\tau \right)\\ & = & g^{2}\tau \left[{\langle H_{k+1}\rangle }_{\mathrm{anc}},\left[{\langle H_{k-m+1}\rangle }_{\mathrm{anc}},\varrho_{S}\right]\right]-g^{2}\tau {\langle \left[H_{k+1},\left[H_{k-m+1},\varrho_{S}⊗I_{\mathrm{anc}}\right]\right]\rangle }_{\mathrm{anc}}+o\left(g^{2}\tau \right). \end{array}$$

Equation (54) provides an important physical link between the two-point correlation function of ancillas and the memory kernel.

## 8. Stroboscopic Limit

To simplify the analysis, let us assume that the correlated ancillas are initially in the homogeneous right-canonical MPDO, i.e., the tensors $B^{\left[k\right]}$ coincide for all k=1,…,n, and MPDO is fully described by the density matrix $\chi_{0}$ and the tensor *M*. In this case, all local density operators for individual ancillas coincide $\varrho_{1}=\ldots =\varrho_{n}$; however, the two-ancilla density operator $\varrho_{12}\neq \varrho_{1}⊗\varrho_{2}$. If ancillas are initially in such a homogeneous state, we can expect that the kernel $K_{km}$ depends on *m* only and does not depend on *k*.

Suppose the collision duration τ tends to zero while the coupling strength *g* remains constant. Then we get the Hamiltonian dynamics for the system $\varrho_{S}\left(t\right)$ in continuous time t=kτ, namely, $\frac{d\varrho_{S}\left(t\right)}{dt}=-ig\left[{\langle H\rangle }_{\mathrm{anc}},\varrho_{S}\left(t\right)\right]$ [66]. The correlations among ancillas are irrelevant in this scenario because $g^{2}\tau \to 0$ in the considered limit.

To reveal a nonunitary system dynamic at a long timescale, one should consider a different limit gτ→0, $g^{2}\tau =\mathrm{const}$ [56,65,66], which we refer to as the (first-order) stroboscopic limit that is also used in the analysis of dynamics induced by indirect repeated measurements [106,107]. The Hamiltonian part $-ig[{\langle H\rangle }_{\mathrm{anc}},\varrho_{S}\left(t\right)]$ explodes in the master equation because g→∞; however, this problem disappears in a proper interaction picture [66].

In the stroboscopic limit, one cannot simply replace $\frac{1}{\tau }\left[\varrho_{S}\left((k+1)\tau \right)-\varrho_{S}\left(k\tau \right)\right]$ in the left hand side of Equation (36) by $\frac{d\varrho_{S}\left(t\right)}{dt}$ if the term $-ig[{\langle H\rangle }_{\mathrm{anc}},\varrho_{S}\left(t\right)]$ does not vanish, because $\frac{1}{\tau }\left[\varrho_{S}\left((k+1)\tau \right)-\varrho_{S}\left(k\tau \right)\right]=\frac{d\varrho_{S}\left(t\right)}{dt}+\frac{\tau }{2}\frac{d^{2}\varrho_{S}\left(t\right)}{dt^{2}}+\ldots =\frac{d\varrho_{S}\left(t\right)}{dt}+O\left(g^{2}\tau \right)$, and the second summand cannot be neglected. However, if the expression $-ig[{\langle H\rangle }_{\mathrm{anc}},\varrho_{S}\left(t\right)]$ vanishes, then the characteristic frequency of system dynamics is $g^{2}\tau$ so that $\frac{1}{\tau }\left[\varrho_{S}\left((k+1)\tau \right)-\varrho_{S}\left(k\tau \right)\right]=\frac{d\varrho_{S}\left(t\right)}{dt}+O\left(g^{4}\tau^{3}\right)$. The second summand vanishes in the (first-order) stroboscopic limit because $g^{4}\tau^{3}={\left(g^{2}\tau \right)}^{2}\tau \to 0$. The time-local memory kernel component should also be considered in this limit, i.e.,

(55) $$\frac{{\mathrm{tr}}_{1}\left[U\varrho_{S}⊗\varrho_{1}U^{†}\right]-\varrho_{S}}{\tau }\to L_{\mathrm{local}}\left[\varrho_{S}\right]\;\mathrm{when}\;g\tau \to 0,g^{2}\tau =\mathrm{const}.$$

The higher-order contributions $K_{km}^{\left(3\right)},K_{km}^{\left(4\right)},\ldots$ to the memory kernel (43) vanish only if the correlation length $l_{\mathrm{corr}}$ (in the chain of ancillas) is finite. If this is the case, then $∥\sum_{m=0}^{k}K_{km}^{\left(N\right)}\left[\varrho_{S}\left((k-m)\tau \right)\right]∥≲l_{\mathrm{corr}}g^{N+1}\tau^{N}\to 0$ for N=3,4,….

Therefore, in the first-order stroboscopic limit, we get the time-continuous master equation

(56) $$\begin{array}{ccc} & & \frac{d\varrho_{S}\left(t\right)}{dt}=\int_{0}^{t}K\left(t^{\prime }\right)\left[\varrho_{S}\left(t-t^{\prime }\right)\right]dt^{\prime }, \end{array}$$

(57) $$\begin{array}{ccc} & & K\left(t^{\prime }\right)\left[\varrho_{S}\right]=\delta \left(t^{\prime }\right)L_{\mathrm{local}}\left[\varrho_{S}\right]+g^{2}\tau \lim_{\tau \to 0}\sum_{m=1}^{\infty }\delta \left(t^{\prime }-m\tau \right)K_{m}\left[\varrho_{S}\right], \end{array}$$

(58) $$\begin{array}{ccc} & & K_{m}\left[\varrho_{S}\right]=\left[{\langle H\rangle }_{\mathrm{anc}},\left[{\langle H\rangle }_{\mathrm{anc}},\varrho_{S}\right]\right]-{\langle \left[H_{m+1},\left[H_{1},\varrho_{S}⊗I_{\mathrm{anc}}\right]\right]\rangle }_{\mathrm{anc}}. \end{array}$$

The correlations are known to decay exponentially in an MPS and an MPDO [73,74,75,76,77], with the correlation length $l_{\mathrm{corr}}$ being defined by the second-largest eigenvalue of the transfer matrix $T=\sum_{i}M^{ii}$ (in absolute values). If the correlation length is finite, then $K_{m}$ represents a sum of exponentially decaying terms,

(59) $$K_{m}\left[\varrho_{S}\right]=\sum_{j}{\left(\lambda_{j}\right)}^{m}L_{\mathrm{nonlocal}}^{\left(j\right)}\left[\varrho_{S}\right],$$

where ${\left\{\lambda_{j}\right\}}_{j}$ are eigenvalues of the transfer matrix *T* such that $|\lambda_{j}|<1$ and $\left\{L_{\mathrm{nonlocal}}^{\left(j\right)}\right\}$ are the corresponding maps.

To explicitly find the kernel $K(t^{\prime })$ in Equation (56), we resort to the Laplace transform (which is often used for the memory kernel master equations [108,109,110]); namely,

(60) $$\begin{array}{ccc} K_{s} & = & \int_{0}^{\infty }K\left(t^{\prime }\right)e^{-st^{\prime }}dt^{\prime }=L_{\mathrm{local}}+g^{2}\tau \lim_{\tau \to 0}\sum_{m=1}^{\infty }e^{-s\tau m}K_{m}\\ & = & L_{\mathrm{local}}+g^{2}\tau \lim_{\tau \to 0}\sum_{j}\left(\sum_{m=1}^{\infty }e^{-s\tau m}{\left(\lambda_{j}\right)}^{m}\right)L_{\mathrm{nonlocal}}^{\left(j\right)}\\ & = & L_{\mathrm{local}}+g^{2}\tau \lim_{\tau \to 0}\sum_{j}\frac{e^{-s\tau }\lambda_{j}}{1-e^{-s\tau }\lambda_{j}}L_{\mathrm{nonlocal}}^{\left(j\right)}\\ & = & L_{\mathrm{local}}+g^{2}\tau \sum_{j}\frac{\lambda_{j}}{1-\lambda_{j}}L_{\mathrm{nonlocal}}^{\left(j\right)}. \end{array}$$

The result does not depend on *s*, which means the kernel $K(t^{\prime })$ becomes local in the stroboscopic limit and the final master equation takes the form

(61) $$\frac{d\varrho_{S}\left(t\right)}{dt}=L_{\mathrm{local}}\left[\varrho_{S}\left(t\right)\right]+g^{2}\tau \sum_{j}\frac{\lambda_{j}}{1-\lambda_{j}}L_{\mathrm{nonlocal}}^{\left(j\right)}\left[\varrho_{S}\left(t\right)\right].$$

Physically, Equation (61) shows that if the system quickly interacts with ancillas (gτ≪1), then the system “feels” not only the individual ancillas (which results in the local term $L_{\mathrm{local}}$) but also a somewhat averaged correlated state (which results in the nonlocal term $g^{2}\tau \sum_{j}\frac{\lambda_{j}}{1-\lambda_{j}}L_{\mathrm{nonlocal}}^{\left(j\right)}$). We summarize these results as follows.

**Proposition** **4.** *Let the system collisionally interact with an array of ancillas in the homogeneous MPDO with a finite correlation length. If the expression $-ig[{\langle H\rangle }_{\mathrm{anc}},\varrho_{S}\left(t\right)]$ vanishes, then in the first-order stroboscopic limit gτ→0, $g^{2}\tau =\mathrm{const}$, the system dynamics are governed by the master equation (61), where the local and nonlocal contributions to the generator are defined by Equations (55), (58) and (59).*

**Example** **5.** *Consider an infinite chain of spin-1 particles (ancillas) in the AKLT state [70] that adopts the following homogenous right canonical MPS representation with the MPS rank 2 [75]:*

(62) $$A^{[k],1}=\left(\begin{array}{cc} 0 & \sqrt{\frac{2}{3}}\\ 0 & 0 \end{array}\right),\;A^{[k],2}=\left(\begin{array}{cc} -\frac{1}{\sqrt{3}} & 0\\ 0 & \frac{1}{\sqrt{3}} \end{array}\right),\;A^{[k],3}=\left(\begin{array}{cc} 0 & 0\\ -\sqrt{\frac{2}{3}} & 0 \end{array}\right).$$

*Each individual ancilla has a reduced density operator $\varrho_{1}=\frac{1}{3}I$; however, the global state is correlated.*

*At time t=0, the qubit system collides with one of the intermediate ancillas, then collides with its right neighbor and so on. Each collision lasts time τ. The system-particle interaction Hamiltonian is*

(63) $$\hbar gH=\hbar g\sum_{j=1,2,3}\sigma_{j}⊗|j\rangle \langle j|.$$

*Averaging over single-ancilla degrees of freedom yields ${\langle H\rangle }_{\mathrm{anc}}=\frac{1}{3}\sum_{j=1,2,3}\sigma_{j}\neq 0$. To use Proposition 4, we set $\varrho_{S}\left(0\right)=\frac{1}{2}\left(I+\frac{1}{\sqrt{3}}\sum_{j=1,2,3}\sigma_{j}\right)$, so that $-ig[{\langle H\rangle }_{\mathrm{anc}},\varrho_{S}\left(0\right)]=0$. As we will see later, the latter commutation relation remains valid for all times t, i.e., $-ig[{\langle H\rangle }_{\mathrm{anc}},\varrho_{S}\left(t\right)]=0$, and the use of Proposition 4 is justified.*

*The local term is given by formula (55) and reads*

(64) $$L_{\mathrm{local}}\left[\varrho_{S}\right]=\frac{g^{2}\tau }{3}\sum_{j=1,2,3}\left(\sigma_{j}\varrho_{S}\sigma_{j}-\varrho_{S}\right).$$

*To find the nonlocal term, we should take correlations into account. Since the system interacts with a part of the infinite spin chain, the state $\varrho_{1\ldots \infty }$ of ancillas (spin-1 particles) is mixed and described by a right-canonical homogeneous MPDO with*

(65) $$\chi_{0}=\frac{1}{2}\left(\begin{array}{cc} 1 & 0\\ 0 & 1 \end{array}\right),\;B_{1}^{[k],1}=\left(\begin{array}{cc} 0 & \sqrt{\frac{2}{3}}\\ 0 & 0 \end{array}\right),\;B_{1}^{[k],2}=\left(\begin{array}{cc} -\frac{1}{\sqrt{3}} & 0\\ 0 & \frac{1}{\sqrt{3}} \end{array}\right),\;B_{1}^{[k],3}=\left(\begin{array}{cc} 0 & 0\\ -\sqrt{\frac{2}{3}} & 0 \end{array}\right).$$

*Note that $M^{ii^{\prime }}=B_{1}^{[k],i}⊗\bar{B_{1}^{\left[k\right],i^{\prime }}}$. The transfer matrix reads*

(66) $$T=\sum_{i}M^{ii}=\frac{1}{3}\left(\begin{array}{cccc} 1 & 0 & 0 & 2\\ 0 & -1 & 0 & 0\\ 0 & 0 & -1 & 0\\ 2 & 0 & 0 & 1 \end{array}\right)$$

*and has eigenvalues 1 (of multiplicity 1) and $-\frac{1}{3}$ (of multiplicity 3). The two-spin reduced density matrix reads*

(67) $$\varrho_{1m}=\frac{1}{3}I⊗\frac{1}{3}I+{\left(-\frac{1}{3}\right)}^{m}\left(J_{x}⊗J_{x}+J_{y}⊗J_{y}+J_{z}⊗J_{z}\right),$$

*where $J_{\alpha }$ is an operator for the spin projection (in units of ℏ) in the α direction, α=x,y,z; see Equation (22).*

*Substituting Equation (67) in Equation (58), we get*

(68) $$K_{m}\left[\varrho_{S}\right]=4{\left(-\frac{1}{3}\right)}^{m+1}g^{2}\tau \left[\frac{\sigma_{x}-\sigma_{z}}{\sqrt{2}}\varrho_{S}\frac{\sigma_{x}-\sigma_{z}}{\sqrt{2}}-\varrho_{S}\right].$$

*On the other hand, $K_{m}\left[\varrho_{S}\right]=\sum_{j}{\left(\lambda_{j}\right)}^{m}L_{\mathrm{nonlocal}}^{\left(j\right)}\left[\varrho_{S}\right]$, i.e., in our case we have a single contribution with $\lambda =-\frac{1}{3}$ and*

(69) $$L_{\mathrm{nonlocal}}\left[\varrho_{S}\right]=-\frac{4}{3}\left(\frac{\sigma_{x}-\sigma_{z}}{\sqrt{2}}\varrho_{S}\frac{\sigma_{x}-\sigma_{z}}{\sqrt{2}}-\varrho_{S}\right).$$

*Finally, Equation (61) gives the explicit master equation in the stroboscopic limit*

(70) $$\frac{d\varrho_{S}\left(t\right)}{dt}=\frac{g^{2}\tau }{3}\sum_{j=1,2,3}\left(\sigma_{j}\varrho_{S}\left(t\right)\sigma_{j}-\varrho_{S}\left(t\right)\right)-\frac{g^{2}\tau }{3}\left(\frac{\sigma_{x}-\sigma_{z}}{\sqrt{2}}\varrho_{S}\left(t\right)\frac{\sigma_{x}-\sigma_{z}}{\sqrt{2}}-\varrho_{S}\left(t\right)\right).$$

**Figure 10:**
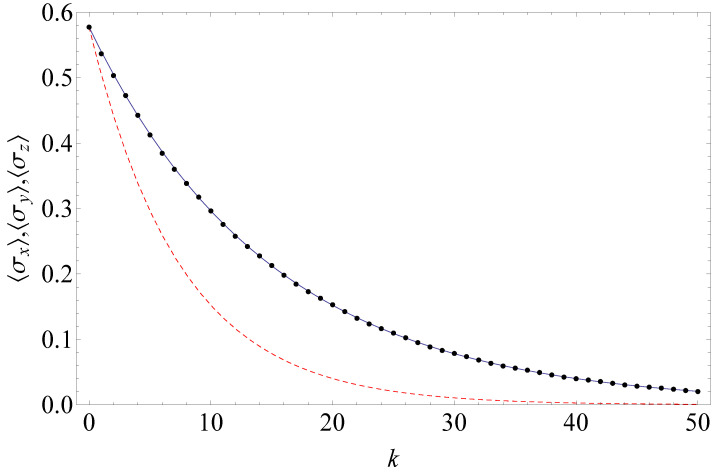
The qubit dynamics in the collision model with correlated ancillas in the AKLT state (Example 5): Bloch vector components $\langle \sigma_{x}\rangle ,\langle \sigma_{y}\rangle ,\langle \sigma_{z}\rangle$ vs. the number of collisions *k*. The initial qubit state is $\varrho_{S}\left(0\right)=\frac{1}{2}\left(I+\frac{1}{\sqrt{3}}\sum_{j=x,y,z}\sigma_{j}\right)$. The system-ancilla Hamiltonian is given by Equation (63). The interaction strength gτ=0.1. The exact solution via Equation (18) is shown by dots. The solution in the stroboscopic limit [Equation (70)] corresponds to a solid line. Disregard of correlations among ancillas leads to the equation $\frac{d\varrho_{S}\left(t\right)}{dt}=L_{\mathrm{local}}\left[\varrho_{S}\left(t\right)\right]$; its solution is shown by a dashed line.

*The reader may notice the formally negative rate $-\frac{g^{2}\tau }{3}$ in front of the second dissipator term; however, the total generator does have the Gorini–Kossakowski–Sudarshan–Lindblad form [111,112] because the Kossakowski matrix is positive semidefinite. Therefore, the actual relaxation rates are positive. The effect of the formally negative rate $-\frac{g^{2}\tau }{3}$ in front of the second dissipator term in Equation (70) is that correlations among ancillas slow down the relaxation as compared to the case of uncorrelated ancillas (equation $\frac{d\varrho_{S}\left(t\right)}{dt}=L_{\mathrm{local}}\left[\varrho_{S}\left(t\right)\right]$). Figure 10 illustrates this phenomenon. Figure 10 also shows a good agreement between the exact system dynamics and the system dynamics in the stroboscopic limit and emphasizes the role of correlations. Disregard of correlations among ancillas leads to a wrong result (see the dashed line in Figure 10).*

## 9. Effect of Multipoint Correlations in the Higher-Order Stroboscopic Limit

We start with an example stimulating the discussion of the higher-order stroboscopic limit.

**Example** **6.** *Consider a qubit system interacting with an infinite chain of spin-1 particles (ancillas) in the AKLT state as in Example 5, with the only difference that the system-ancilla interaction Hamiltonian now reads*

(71) $$\hbar gH=\frac{\hbar g}{2}\sum_{j=x,y,z}\sigma_{j}⊗J_{j}.$$

*For such an interaction, ${\langle H\rangle }_{\mathrm{anc}}=0$, so the use of Proposition 4 is justified. Following the lines of Example 5, we similarly calculate $L_{\mathrm{local}}$ and $L_{\mathrm{nonlocal}}$ in the first-order stroboscopic limit; however, these contributions cancel each other so that the right hand side of Equation (61) vanishes in the first-order stroboscopic limit, and we get $\frac{d\varrho_{S}\left(t\right)}{dt}=0$.*

*The exact treatment of the problem via Equation (18) yields the depolarizing system dynamics*

(72) $$\varrho_{S}\left(t\right)=q\left(t\right)\varrho_{S}\left(0\right)+\left[1-q\left(t\right)\right]\frac{1}{2}I$$

*with the depolarization function*

(73) $$\begin{array}{ccc} & & q\left(t\right)=\left(\frac{1}{2}+\frac{x}{z}\right){\left(\frac{y+z}{27}\right)}^{t/\tau }+\left(\frac{1}{2}-\frac{x}{z}\right){\left(\frac{y-z}{27}\right)}^{t/\tau }, \end{array}$$

(74) $$\begin{array}{ccc} & & x=2+7\cos\frac{3g\tau }{2},\;y=7+2\cos\frac{3g\tau }{2},\;z=2\sqrt{y^{2}+27\sin^{2}\frac{3g\tau }{2}}. \end{array}$$

*A feature of the depolarizing dynamical map (72) is that it is neither completely positive divisible nor positive divisible for all $g\tau \leq \frac{2}{3}\arccos\left(-\frac{11}{16}\right)$ because*

(75) $$q\left(\tau \right)\geq 0\;\mathrm{and}\;q\left(2\tau \right)-q\left(\tau \right)=\frac{2^{5}y}{3^{6}}\sin^{2}\frac{3g\tau }{4}>0,$$

*i.e., the image of the system Bloch ball shrinks after the first collision and then expands after the second collision. If gτ=4πm/3, m∈N, then the system experiences no evolution, i.e., $\varrho_{S}\left(t\right)=\varrho_{S}\left(0\right)$. If gτ=2π/3, then the Bloch ball experiences partial inversion with the scaling parameter $-\frac{5}{27}$ after each collision. The latter dynamics are, however, completely positive divisible.*

*If gτ≪1, then $q\left(t\right)\approx \exp\left(-\frac{1}{8}g^{4}\tau^{3}t\right)$, so the characteristic frequency of the system dynamics is $g^{4}\tau^{3}$. The first-order stroboscopic limit is unable to reproduce such a behaviour because it is only sensitive to rates $∼g^{2}\tau$. This example stimulates us to develop (to some extent) the theory of the higher-order stroboscopic limit.*

We will refer to the limit gτ→0, $g^{n+1}\tau^{n}=\mathrm{const}$ as the *n*-th order stroboscopic limit. Surely, the expressions *g*, $g^{2}\tau$, …, $g^{n}\tau^{n-1}$ explode in this limit; however, if their contribution to the system dynamics vanishes, then the limit is well defined. The higher-order contributions $g^{n+2}\tau^{n+1}$, $g^{n+3}\tau^{n+2}$, …vanish in the *n*-th order stroboscopic limit. In Equation (55), for $L_{\mathrm{local}}$, we should throw away the exploding terms (as they will cancel other exploding terms from the memory kernel) and vanishing terms. In the memory kernel series expansion (43), we should keep the term $K^{\left(n+1\right)}$, which describes (n+1)-point correlations among ancillas. Recalling the notation in Equation (41), we get, for instance, the following expression for the third-order memory-kernel:

(76) $$\begin{array}{ccc} K_{km}^{\left(3\right)}\left[\varrho_{S}\right] & = & \sum_{i_{k+1},i_{k+1}^{\prime },i_{k-m+1},i_{k-m+1}^{\prime }}C_{i_{k-m+1}i_{k-m+1}^{\prime }i_{k+1}i_{k+1}^{\prime }}^{\left(2\right)}\left(\Phi_{i_{k+1}i_{k+1}^{\prime }}^{[k+1],\left(1\right)}\circ \Phi_{i_{k-m+1}i_{k-m+1}^{\prime }}^{[k-m+1],\left(2\right)}\left[\varrho_{S}\right]+\Phi_{i_{k+1}i_{k+1}^{\prime }}^{[k+1],\left(2\right)}\circ \Phi_{i_{k-m+1}i_{k-m+1}^{\prime }}^{[k-m+1],\left(1\right)}\left[\varrho_{S}\right]\right)\\ & & +\sum_{l=k-m+2}^{k}\;\sum_{i_{k+1},i_{k+1}^{\prime },i_{l},i_{l}^{\prime },i_{k-m+1},i_{k-m+1}^{\prime }}C_{i_{k-m+1}i_{k-m+1}^{\prime }i_{l}i_{l}^{\prime }i_{k+1}i_{k+1}^{\prime }}^{\left(3\right)}\Phi_{i_{k+1}i_{k+1}^{\prime }}^{[k+1],\left(1\right)}\circ \Phi_{i_{l}i_{l}^{\prime }}^{[l],\left(1\right)}\circ \Phi_{i_{k-m+1}i_{k-m+1}^{\prime }}^{[k-m+1],\left(1\right)}\left[\varrho_{S}\right], \end{array}$$

where $C_{i_{k-m+1}i_{k-m+1}^{\prime }i_{l}i_{l}^{\prime }i_{k+1}i_{k+1}^{\prime }}^{\left(3\right)}$ is a three-point correlation function, which is determined by tensor diagrams similar to those in Figure 9c and reads

(77) $$\begin{array}{ccc} & & C_{i_{k-m+1}i_{k-m+1}^{\prime }i_{l}i_{l}^{\prime }i_{k+1}i_{k+1}^{\prime }}^{\left(3\right)}=C\left(|i_{k-m+1}^{\prime }\rangle \langle i_{k-m+1}|,\;|i_{l}^{\prime }\rangle \langle i_{l}|,\;|i_{k+1}^{\prime }\rangle \langle i_{k+1}|\right), \end{array}$$

(78) $$\begin{array}{ccc} & & C\left(O,O^{\prime },O^{\prime \prime }\right)\equiv {\langle OO^{\prime }O^{\prime \prime }\rangle }_{\mathrm{anc}}-{\langle OO^{\prime }\rangle }_{\mathrm{anc}}{\langle O^{\prime \prime }\rangle }_{\mathrm{anc}}-{\langle O\rangle }_{\mathrm{anc}}{\langle O^{\prime }O^{\prime \prime }\rangle }_{\mathrm{anc}}+{\langle O\rangle }_{\mathrm{anc}}{\langle O^{\prime }\rangle }_{\mathrm{anc}}{\langle O^{\prime \prime }\rangle }_{\mathrm{anc}}, \end{array}$$

with $C(O,O^{\prime },O^{\prime \prime })$ being the third-order Waldenfelds cumulant [113,114]. In the second-order stroboscopic limit, the expression $g^{3}\tau^{2}K_{km}^{\left(3\right)}$ reduces to the time-local generator $g^{3}\tau^{2}L_{\mathrm{nonlocal}}$, which contributes to the final GKSL master equation $\frac{d\varrho_{S}\left(t\right)}{dt}=L_{\mathrm{local}}\left[\varrho_{S}\left(t\right)\right]+g^{3}\tau^{2}L_{\mathrm{nonlocal}}\left[\varrho_{S}\left(t\right)\right]$. The higher-order Waldenfelds cumulants are expressed through the lower-order ones [114], thus enabling one to achieve a desired stroboscopic order. To correctly describe evolution in Example 6, one needs to consider the third-order stroboscopic limit.

To give a broader view on the achieved result, the language of tensor networks enabled us to relate the memory kernel components with the multipoint correlation functions of the special form (the Waldenfelds cumulants). Multipoint correlations of orders *n*, n−1, …, 2 determine the system dynamics in the (n−1)-th order stroboscopic limit. Although multitime correlation functions have been used in the theory of open quantum systems (see, e.g., [115,116,117]), here we have explicitly demonstrated their origin in the collision model. We believe that the tensor network representation opens an avenue for a further analysis of the effect of multipoint correlations on the collisional dynamics, e.g., Wick’s theorem for matrix product states [118] can be of great use.

## 10. Conclusions

We presented a tensor network approach to challenges in both the standard collision model and the collision model with correlated ancillas. We showed that the system-ancilla interactions in the standard collision model induce a correlated state of the system and ancillas that is naturally described by a right-canonical MPS (if the system and ancillas are initially in pure states) and a right-canonical MPDO (if the system and ancillas are initially in mixed states). Since the description of MPS and MPDO requires many fewer parameters as compared to a general multipartite state, we believe that the revealed representation can find applications in many practically relevant problems, e.g., this representation can allow one to go well beyond 12 collisions in the numerical study of quantum thermometry [34]. As far as initially correlated ancillas are concerned, we reviewed the recently proposed approach to the tensor network description of the system dynamics (with the emphasis on the two-point correlations) and generalized it to the case of multipartite correlations among ancillas. We showed conditions under which the higher-order stroboscopic limit is to be considered and how the Waldenfelds cumulants contribute to the memory-kernel master equation in this case.

## Data Availability

The data presented in this study are available in article.
